# Medicinal Plant Extracts and Natural Compounds for the Treatment of Cutaneous Lupus Erythematosus: A Systematic Review

**DOI:** 10.3389/fphar.2022.802624

**Published:** 2022-03-31

**Authors:** Janet E. Lubov, Aisha S. Jamison, Becky Baltich Nelson, Alice A. Amudzi, Kelly N. Haas, Jillian M. Richmond

**Affiliations:** ^1^ Department of Dermatology, UMass Chan Medical School, Worcester, MA, United States; ^2^ Wright State University Boonshoft School of Medicine, Dayton, OH, United States; ^3^ Lamar Soutter Library, UMass Chan Medical School, Worcester, MA, United States; ^4^ Department of Microbiology, UMass Amherst, Amherst, MA, United States

**Keywords:** cutaneous lupus erythematosus (CLE), natural compounds, medicinal plant extracts, traditional Chinese medicine, herbal formulas, medicinal mushroom extracts, anti-inflammatory, skin disease

## Abstract

Cutaneous lupus erythematosus (CLE) is a group of autoimmune connective tissue disorders that significantly impact quality of life. Current treatment approaches typically use antimalarial medications, though patients may become recalcitrant. Other treatment options include general immunosuppressants, highlighting the need for more and more targeted treatment options. The purpose of this systematic review was to identify potential compounds that could be repurposed for CLE from natural products since many rheumatologic drugs are derived from natural products, including antimalarials*.* This study was registered with PROSPERO, the international prospective register of systematic reviews (registration number CRD42021251048). We comprehensively searched Ovid Medline, Cochrane Library, and Scopus databases from inception to April 27th, 2021. These terms included cutaneous lupus erythematosus; general plant, fungus, bacteria terminology; selected plants and plant-derived products; selected antimalarials; and JAK inhibitors. Our search yielded 13,970 studies, of which 1,362 were duplicates. We screened 12,608 abstracts, found 12,043 to be irrelevant, and assessed 565 full-text studies for eligibility. Of these, 506 were excluded, and 59 studies were included in the data extraction. The ROBINS-I risk of bias assessment tool was used to assess studies that met our inclusion criteria. According to our findings, several natural compounds do reduce inflammation in lupus and other autoimmune skin diseases in studies using *in vitro* methods, mouse models, and clinical observational studies, along with a few randomized clinical trials. Our study has cataloged evidence in support of potential natural compounds and plant extracts that could serve as novel sources of active ingredients for the treatment of CLE*.* It is imperative that further studies in mice and humans are conducted to validate these findings.

**Systematic Review Registration:** https://www.crd.york.ac.uk/prospero/display_record.php?RecordID=251048.

## 1 Introduction

Cutaneous lupus erythematosus (CLE) is a spectrum of autoimmune connective tissue disorders that causes a significant health burden. In addition to being a symptom of systemic lupus erythematosus (SLE), CLE can exist as its entity. Patients report negative impacts on mental health, employment, and overall health (Klein et al., 2011; Ogunsanya et al., 2020). CLE can be triggered by exposure to UV light, and photosensitivity can trigger flares both in the skin and systemically. Sun avoidance also impacts the quality of life, and patients often require vitamin D supplementation. Controlling skin disease can prevent SLE flares, providing further evidence of a link between the skin, the immune system, and other organs in the body. The most recent FDA-approved treatments for SLE were Benlysta (belimumab) in 2011 and Saphnelo (anifrolumab-fnia) in 2021. There is a need for more treatment options to address the clinical and immunological heterogeneity of lupus and CLE.

**FIGURE 1:**
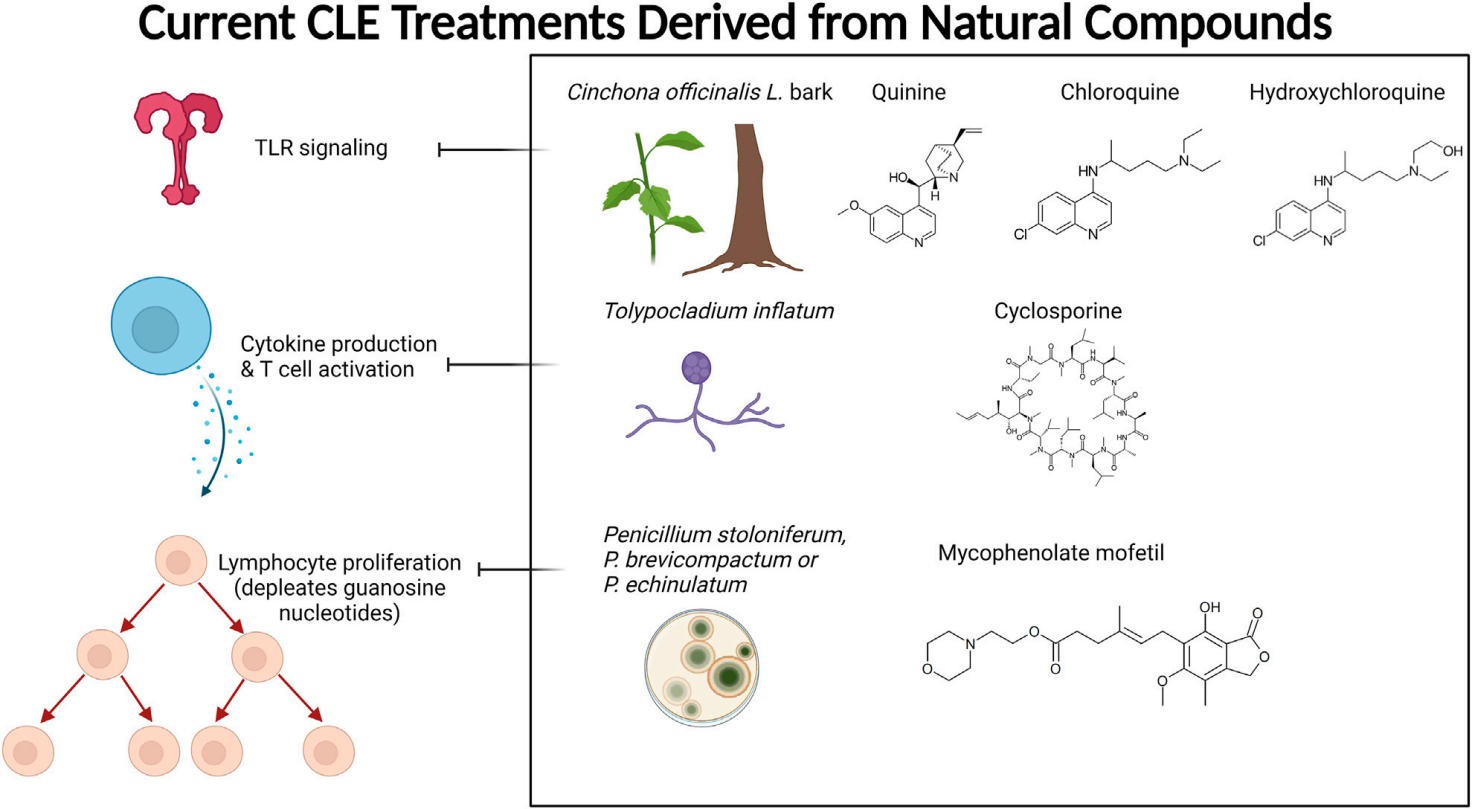
Current CLE treatments derived from natural compounds. Antimalarials are originally derived from the bark of the Cinchona tree. The active ingredient quinine is a TLR inhibitor that provides relief and maintenance therapy for CLE patients. Cyclosporine A (CsA) is also used to treat CLE, which is derived from the fermentation of the fungus *Tolypocladium inflatum* and related species. CsA prevents cytokine production (mainly IL-2) and subsequent T cell activation. Mycophenolate mofetil (CellCept) is also fungal-derived from *Penicillium* species, and its mechanism of action is to deplete guanosine nucleotides preferentially in T and B lymphocytes, which ultimately prevents proliferation, antibody formation, and cell-mediated immunity. Created with BioRender.com.

Antimalarial compounds, which are used to treat the majority of CLE patients (Hejazi and Werth, 2016), are plant-derived. Quinine from Cinchona tree (*Cinchona officinalis L. [*Rubiaceae*]*) bark extract was used by Peruvian peoples to treat fevers. Europeans determined how to create synthetic derivatives, including chloroquine and hydroxychloroquine (Plaquenil), which are used today to treat systemic and cutaneous lupus patients (Achan et al., 2011). Similarly, other successful treatments for rheumatologic and autoimmune skin conditions, including SLE and CLE, are purified from, or synthetic derivatives of, naturally occurring compounds. Mycophenolate mofetil (CellCept), which is used to treat discoid lupus in addition to several other conditions, is derived from the fungi *Penicillium stoloniferum, P. brevicompactum,* and *P. echinulatum* (Allison, 2005). Cyclosporine A is used to treat rheumatoid arthritis and psoriasis, and is produced by fermentation of the fungus *Trichoderma polysporum* (currently identified as *Tolypocladium inflatum*) (Kuhn et al., 2011). Tacrolimus (FK 506), a calcineurin inhibitor used to treat skin conditions including CLE, is derived from the soil bacterium *Streptomyces tsukubaensis* (Sárdy et al., 2009). Figure 1 summarizes the currently used CLE treatments derived from natural products and their targets. Other rheumatologic conditions have been treated with folk medicine (Salmón, 2020), herbalism (Yarnell and Abascal, 2008), and Traditional Chinese Medicine (TCM) (Chang et al., 1997; Ma et al., 2016; Wang Y. et al., 2021).

Based on the previous successes of natural compounds for the treatment of rheumatologic conditions, we hypothesized that other plant extracts and natural compounds could provide active ingredients that could be developed specifically for CLE treatment. To this end, our objective was to perform a systematic review of medicinal plant extracts and natural compounds to identify those with demonstrated efficacy for lupus and/or skin diseases that are not currently used to treat CLE. Our long-term goal is to repurpose these compounds for the treatment of CLE.

## 2 Methods

### 2.1 Protocol and Registration

This study was registered with PROSPERO, the international prospective register of systematic reviews (registration number CRD42021251048), and followed the guidelines set forth by the Preferred Reporting Items for Systematic Reviews and Meta-Analyses (PRISMA) statement (Liberati et al., 2009).

### 2.2 Search Strategy

A comprehensive literature search was conducted by a medical librarian on April 27th, 2021, using the following bibliographic databases from inception: Ovid MEDLINE^®^ (ALL-1946 to Present); Cochrane Library (Wiley); and Scopus (Elsevier). No article type, date, or language restrictions were included in the search. Controlled vocabulary and keywords for cutaneous lupus erythematosus; general plant, fungi, and bacteria terminology; selected plants and plant-derived products; selected antimalarials; and JAK inhibitors. We used adjacency searching to ensure that we would still capture relevant studies even if terms were listed in a different order. The latter search terms were included to capture historical literature about antimalarials from Cinchona tree (*Cinchona officinalis L. [*Rubiaceae*]*) and JAK inhibitors from green tea (*Camellia sinensis (L.) Kuntze*), though ultimately manuscripts that only discussed synthetic derivatives were excluded. The entire Ovid MEDLINE search strategy is available in Supplementary Table 1.

### 2.3 Eligibility Criteria

To be eligible, articles had to meet the following inclusion criteria: 1) studies involving cutaneous lupus erythematosus; 2) studies involving herbalism, plant derivatives, or natural compounds (derived from plants via extraction, expression, or distillation without intentional chemical reaction or modification). Exclusion criteria included: 1) any synthetic products, including any pharmaceutical-grade products that are derivatives of natural products; 2) studies of diseases that were not skin diseases; 3) studies of diseases that were not rheumatologic diseases with related pathogenesis (studies of SLE or related interferonopathies were considered if they met other inclusion criteria); and 4) studies in a language other than English.

Exclusion criteria for full-text review included the following: 1) duplicate article; 2) discusses a natural compound that causes SLE; 3) addressed a natural compound, but not pertinent to CLE or other inclusion criteria; 4) studies involving hydroxychloroquine or related compounds; 5) full article not available in all libraries for interlibrary loan request; 6) full text not in English; 7) did not address specific natural compounds and plant extracts.

#### 2.3.1 Article Types

Article types included from the full-text review were randomized control trials, non-randomized experimental studies, case-control studies, cohort studies, cross-sectional studies, comparative studies, systematic reviews, observational studies, prevalence studies, open-label trials, *in vitro* experiments, *ex vivo* studies, and mouse studies. Review articles, editorials, and text/opinion pieces were excluded in the full-text review.

#### 2.3.2 Participant Types

Studies included human participants with CLE/SLE, human peripheral mononuclear cells of CLE/SLE/healthy patients, lupus-prone rodents, and human keratinocyte cell lines (Refer to Table 1). We included a broad range of study types per our PROSPERO registration due to the fact that this is a very under-studied area and we wanted to capture a large list of potential compounds.

**TABLE 1:** Synthesized findings for potential bioactive compounds for CLE.

Active compound	Effective dose	Source	Experimental system	Mechanism(s) of action/Outcomes	Reference
Vitamins					
Vitamin D (cholecalciferol) 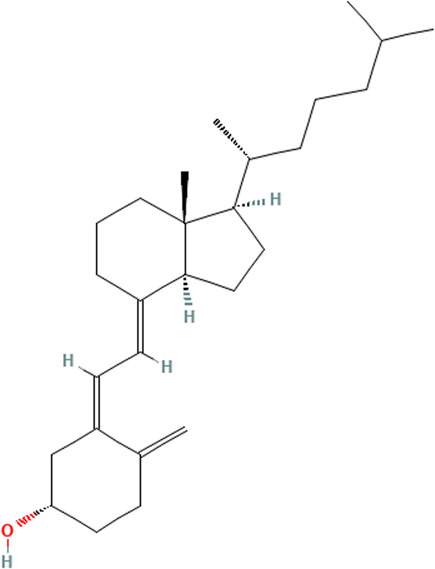	Vitamin D levels <40 nmol/L associated with rise in SLEDAI-2K	Vitamin D	Human Cohort Study on SLE patients	• There was a significant inverse correlation of SLEDAI-2K with baseline vitamin D level and with vitamin D supplementation	Yap et al. (2015)
	N/A	Vitamin D	Human Cross Sectional Study on CLE patients and Healthy African American subjects	• Demonstrated that skin color (African American vs caucasian/Hispanic) had a significant effect on 25-OH vitamin D levels though CLE status (CLE vs normal) did not	Word et al. (2012)
	N/A	Vitamin D	Human Comparative Study on CLE patients	• Vitamin D deficiency in patients with CLE was prevalent throughout the year	Heine et al. (2010)
	1,400 IU of cholecalciferol, plus 1,250 mg of calcium carbon-ate, per day for 40 days, followed by a tablet twice aday of a fixed combination of 1,250 mg of calciumcarbonate and 400 IU of cholecalciferol for 1 year of treatment	Vitamin D	Stage 1: Patients with CLE who were compared to age- and sex-matched unaffected subjects recruited from healthcare workers and adults accompanying the patients who attended clinic in summer 2008	• Presence of CLE raised the odds of having vitamin D deficiency (OR 3.47, 95% CI 1.79-6.69)	Cutillas-Marco et al. (2014)
			Stage 2: CLE patients with vitamin D insufficiency who were treated with and without vitamen D	• Increasing age and disease duration were associated with higher odds of having vitamin D deficiency	
				• Significant improvement in disease activity according to physician and patient assessments	
				• CLASI A decreased from 2.7 ±2.9 to 0.9 ± 1.4 (*p* = 0.003), but CLASI D did not significantly change	
				• There was a consistent trend towards a lower number of exacerbations per year in the treatment group, however this was not statistically significant	
	N/A	Vitamin D	Human Cohort Study on CLE patients	• 25(OH)D levels were significantly lower among sun avoiders and daily sunscreen users, while significantly higher values were found among those who took cholecalciferol (vitamin D3) supplements	Cusack et al. (2008)
				• Low vitamin D values were recorded among patients with renal disease despite supplementation with vitamin D3 in some cases	
	50 nM of 1,25 dihydroxyvitamin D3	Vitamin D	SLE Peripheral blood mononuclear cells (PBMCs)	• Significantly downregulated the expression of TLR3, TRL7, and TRL9	Yazdanpanah et al. (2017)
	N/A	Vitamin D	Patients with CLE attending the outpatient clinics at two dermatology centres in Singapore from October 2009 to February 2013	• Asian CLE patients had significantly lower vitamin D	Gronhagen et al. (2016)
				• No significant differences in (25(OH)D) levels were found between cases and controls regarding age, sex, ethnicity, smoking, sun exposure, sunblock use or vitamin D supplementation	
				• Treatment with antimalarials showed a statistically significant association with lower vitamin D levels	
Oil					
Eicosapentaenoic acid (EPA) 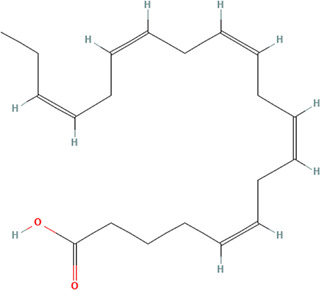	3 g	Fish Oil	Human Randomized Control Trial on SLE patients	A decline in SLAM-R score in those subjects taking fish oil compared to placebo. No significant effect on SLAM-R was observed in subjects taking copper. Laboratory variables were unaffected by either intervention	Duffy, et al.:(2003)
Phenolic Fraction containing (Hydroxytyrosol, Tyrosol,Vanillic acid, P-coumaric acid, Decarboxymethyl oleuropein aglycone (dialdehyde), Tyrosyl acetate, Decarboxymethyl ligstroside aglycone (dialdehyde), Pinoresinol, Cinnamic acid, Acetoxy-pinoresinol, Oleuropein aglycone. aldehyde form, Ligstroside aglycone. dialdehyde form, Luteolin, Apigenin) ---No sigle active ingredient was reported Mostly contains Hydroxytyrosol	10 uM	Phenolic fraction (PE) of Extra Virgin Olive Oil (EVOO)	Human peripheral blood mononuclear cells from SLE patients and healthy controls -- *in vitro*	• ↓ the frequency of CD69^+^ cells and the secretion of IFN-gamma, TNF-alpha, IL-6, IL-1, and IL-10	Aparicio-Soto et al. (2017)
				• ↑ the expression of I-kappa-B on peripheral blood mononuclear cells	
				• ↓ extracellular signal-regulated kinase phosphorylation on peripheral blood mononuclear cells	

Omega 3 Fatty acid and Cholecalcipherol 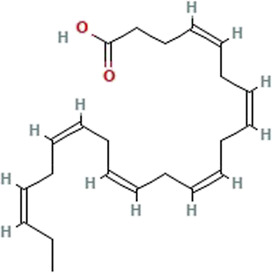 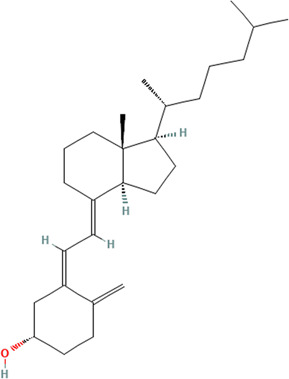	500 ul	Seluang fish oil	Human Randomized Control trial on SLE patients	• The administration of Seluang fish oil was clinically able to show efficacy assessed by the MEX SLEDAI score	Partan et al. (2019)
				• ↑ in vitamin D levels	
				• ↓ in levels of IL-1, IL-6 and IL-17	
Eicosapentaenoic acid (EPA) and Docosahexaenoic acid (DHA) 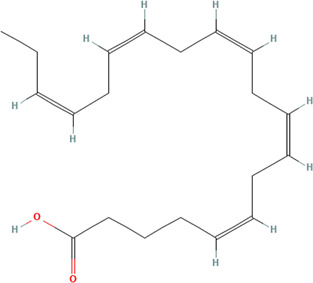 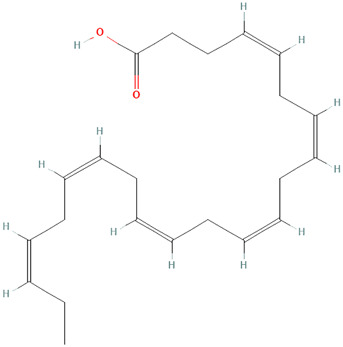	2.25 g EPA and 2.25 g DHA	Fish Oil	Human SLE patients	• Significant improvement of Physician Global Assessment (PGA)	Arriens et al. (2015)
				• Improved RAND SF-36 Energy/fatigue and Emotional well-being scores	
				• No clear difference was seen in Fatigue Severity Scale (FSS)	
				• No clear difference was seen in SLE Disease Activity Index (SLEDAI)	
				• ↓** erythrocyte sedimentation rate and serum IL-12	
				• ↑** serum IL-13	
	1.8 g eicosapenta-noic acid (EPA) and 1.2 g docosahexanoic acid (DHA)	Omega-3-polyunsaturated fatty acids	Human Randomized Control Trial on SLE patients	• ↓ in SLAM-R at 12 weeks and 24 weeks of intervention	Wright et al. (2008)
				• ↓ in the individual scores at 12 weeks for constitutional symptoms and joints and at 24 weeks in constitutional symptoms, integument, neuromotor and joint scores	
				• ↓ BILAG in the general symptoms, cutaneous, musculoskeletal, cardiorespiratory, and vasculitis scores	
Plants					
Aconitine 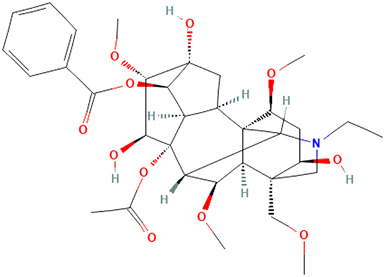	25, 75 μg kg^−1^ day^−1^, po	Aconitum lamarckii Rchb. ex Spreng. [Ranunculaceae]	Female BALB/c mice	↓ Elevated blood leukocyte counts	Li et al. (2017)
				↓ Serum level of anti-double-stranded DNA (anti-dsDNA) antibody	
				↓ IgG deposit in glomerular ameliorating renal histopathology	
				↓ Levels of PGE2, IL-17a and IL-6	
Artemisin compound 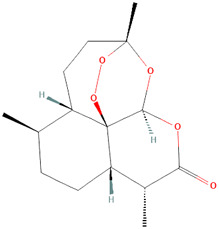	1 mg	Artemisia annua L. [Asteraceae]	Mice and *in vitro* mouse cell assays	↓** Concanavalin A (Con A) and lipopolysaccharide (LPS)-stimulated splenocyte proliferation *in vitro*	Musallam et al. (2009)
				↓** Con A-, LPS- and ovalbumin immunized mice (OVA)-induced splenocyte	
				↓ OVA-specific serum IgG, IgG1 and IgG2b antibody levels in the OVA-immunized mice	
SM934 (beta-Aminoarteether maleate) 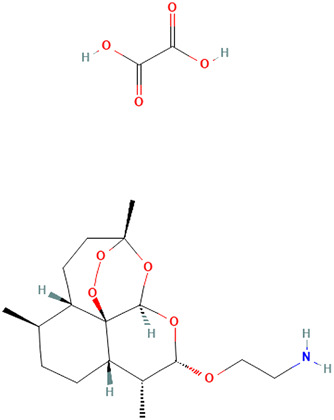	5 mg/kg	Artemisia annua L. [Asteraceae]	Female MRL/lpr mice and PBMCs	• ↑** The lifespan of MRL/lpr mice and ameliorated the lymphadenopathy symptoms	Wu et al. (2016)
				• ↓** Levels of serum anti-nuclear antibodies (ANAs)	
				• ↓** IL-6, IL-10, and IL-21	
				• ↑ Quiescent B cell numbers thereby restoring the B-cell compartment in the spleen of MRL/lpr mice	
				• Maintained germinal center B-cell numbers	
				• ↓ Activated B cell numbers	
				• ↓ Plasma cell (PC) numbers	
				• Suppressed the Toll-like receptor (TLR) *ex-vivo*	
				• Triggered activation and proliferation of B cells and antibody secretion *ex-vivo*	
				• Downregulated TLR7/9 mRNA expression, MyD88 protein expression and NF-κB phosphorylation thereby interfering with the B-cell intrinsic pathway	
				• Inhibited TLR-associated B-cell activation and PC differentiation in human peripheral blood mononuclear cells (PBMCs)	
Celastrol (tripterine) 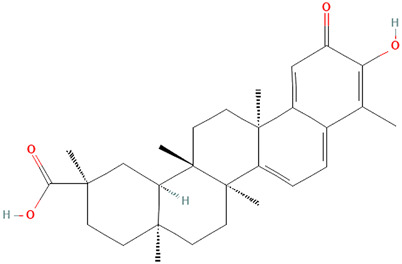	TNFα induced NET - IC50 of 0.34 µM	Tripterygium wilfordii Hook.f. [Celastraceae]	Human PBMCs from patients and controls	• Inhibits neutrophil oxidative burst (NOB) and Neutrophil extracellular traps (NETs) formation induced by tumor necrosis factor alpha (TNFα)	Yu et al. (2015)
	Ova IC induced NET - IC50 of 1.53 µM			• Inhibits NOB and NET formation induced by immunoglobulin G (IgG) purified from RA and SLE patient sera	
				• Downregulates the activation of spleen tyrosine kinase (SYK), mitogen-activated protein kinase kinase (MAPKK/MEK), extracellular-signal-regulated kinase (ERK), and NFκB inhibitor alpha (IκBα), and citrullination of histones	
Curcumin/Turmeric 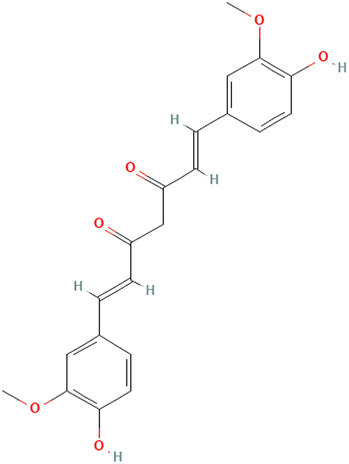	5 mg/ml	Curcuma longa L. [Zingiberaceae]	BALB/c mice	• ↓** binding of autoantibodies to their cognate antigens by up to 70% in systemic lupus erythematosus	Kurien et al. (2010)
				• Inhibition was not specific to autoimmunity	
				• Inhibited binding of commercial polyclonal anti-spectrin to spectrin	
Curcumin 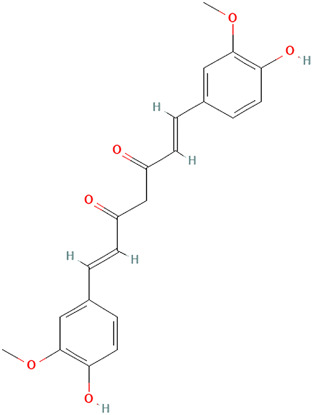	200 mg/kg	Curcuma longa L. [Zingiberaceae]	Female MRL/lpr mice	• ↓ Proteinuria and renal inflammation	Zhao et al. (2019)
				• ↓ Serum anti-dsDNA levels and spleen size	
				• ↓ NLRP3 inflammasome activation in lupus-prone mice	
				• Significantly inhibited anti-dsDNA+ serum induced expression of NLRP3 inflammasome in podocytes *in vitro*	
Curcumin Quercetin 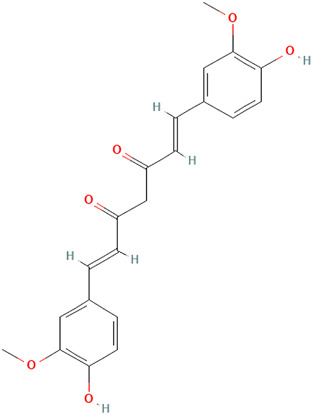 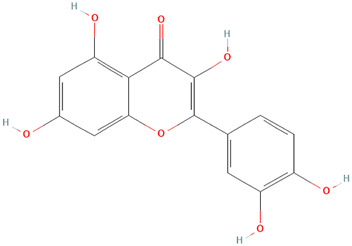	10 mg/ml of Curcumin and Quercetin in each vesicle	Curcumin and Quercetin [Sigma–Aldrich (Milan, Italy)]	Female Hsd:ICR (CD-1) mice	• Quercetin and curcumin nanovesicles in counteracting phorbol ester 12-O-tetradecanoylphorbol-13-acetate (TPA) induced lesions and inflammation *in vitro* and *in vivo*	Castangia et al. (2014)
				• Quercetin liposomes (59%) and curcumin liposomes and polyethylene glycol (PEG)-PEVs (∼ 68%) inhibited myeloperoxydase activity	
				• PEG-PEVs provided an extensive re-epithelization of TPA-damaged skin, with multiple layers of thick epidermis	
Icaritin 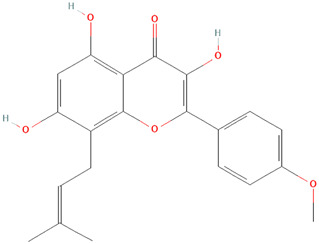	2 mg/kg daily	Epimedium alpinum L. [Berberidaceae]	Human SLE peripheral blood mononuclear cells, MRL/lpr mice	• Regulating Foxp3/IL17a balance by ↑ STAT5b expression and histone methylation modification	Liao et al. (2016)
				• ↑ Treg cell suppressive activities	
				• Inhibited over-activation of CD4 (+) T cells from SLE	
				• Ameliorated renal damages	
No active compound isolated - structure not available	500 mg/body weight (BW)/12 weeks	Camellia sinensis (L.) Kuntze [Theaceae]	C57BL/6 mice	• ↑ energy expenditure of high-fat diet-fed mice (16 weeks)	Otton et al. (2018)
				• ↓ weight gain	
				• ↓ adiposity	
				• ↓ inflammation	
				• ↑ insulin sensitivity	
				• ↓ the expression of miR-335 in the adipose tissue	
				• miR-335 was up-regulated by TNF-α in adipocytes and, in turn, down-regulated genes involved in insulin signaling and lipid metabolism. On the other hand, GT	
				• Inhibited TNF-α up-regulation of miR-335	
No active compound isolated - structure not available	50 and 100 μg/ml	Illicium verum Hook.f. [Schisandraceae]	HaCaT cell line	• Significantly inhibited IFN-γ-induced mRNA and protein expression of ICAM-1	Sung and Kim. (2013)
				• Inhibited IFN-γ-induced IFN-γRα, pJak2 and pSTAT1 expression in HaCaT cells	
				• Up-regulated SOCS1	
				• Suppressed IFN-γ-induced adherence of Jurkat T cells to HaCaT cells and ICAM-1 expression on the cell surface	
Enmein 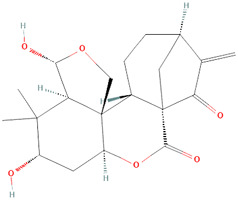	6.25 to 25 µg/ml	Isodon serra (Maxim.) Kudô [Lamiaceae]	BALB/c mice, splenocytes, xylene-induced mouse tumescence?	• Suppressed murine splenic T lymphocytes overproduction stimulated by Concanavalin A	Zhang et al. (2005)
				• ↓ normal sleep lymphocytes than the stimulated ones	
				• Interfered with DNA replication in G1-S stage	
				• Regulated cell cycle	
				• ↓ murine ear swelling extent	
				• ↓ level of IL-2 in blood serum	
Oridonin 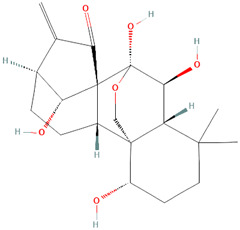	9 mg/kg	Isodon serra (Maxim.) Kudô [Lamiaceae]	MRL/lpr mice and RAW264.7 cells	• ↑ survival benefit *in vivo*	Zhou et al. (2013)
				• ↓ proteinuria levels *in vivo*	
				• ↓ production of specific auto-antibodies	
				• ↓ renal damage	
				• Significantly down-regulated BAFF resulting in a lower rate of B-cell maturation and differentiation	
				• Suppressing the transcriptional activation of BAFF promoter	
Isogarcinol 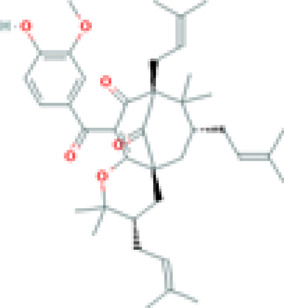	30–60 mg/kg	Garcinia mangostana L. [Clusiaceae]	Female DBA/2 and BDF1 mice	• ↓** proteinuria	Li et al. (2015)
				• ↓ the amount of serum antibodies	
				• ↓ the renal histopathology score	
				• Alleviated the abnormal activation of CD4 T cells	
				• ↓ expression of inflammatory genes and cytokines in the kidneys and peritoneal macrophages	
(S) - Armepavine 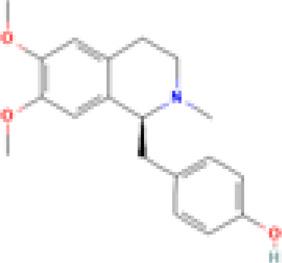	5 and 10 mg/kg/day	Nelumbo nucifera Gaertn. [Nelumbonaceae]	Female MRL/MpJ-lpr/lpr mice and healthy human PBMCs	• ↓expression of IL-2, IL-4, IL-10, and interferon-gamma (IFN-gamma)	Liu et al. (2006)
				• ↓glomerular hypercellularity and immune complexes deposition	
				• ↓urinary protein and anti-double-stranded DNA autoantibody production	
				• Impaired IL-2 and IFN-gamma transcripts in human peripheral blood mononuclear cells (PBMCs)	
				• Prevented lymphadenopathy	
				• Elongated life span of MRL/MpJ-lpr/lpr mice	
				• Inhibited splenocytes proliferation	
Paeoniflorin 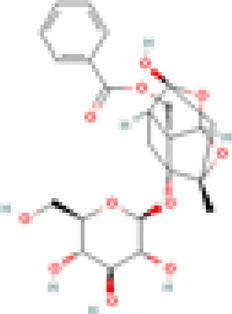	75 or 150 mg/kg/day	Paeonia lactiflora Pall. [Paeoniaceae]	Human *ex vivo* PBMCs, BALB/c mice	• ↓ human T lymphocytes activation via inhibition of interferon-gamma (IFN-γ) production and NF-κB/IκBα and p38 MAPK signaling pathway *in vitro*.	Wu et al. (2021)
				• Inhibited the production of IFN-γ and NF-κB/IκBα pathway in T lymphocytes of Allergic Contact Dermatitis in mouse model	
				• Immunosuppressive and anti-inflammatory	
Paeoniflorin 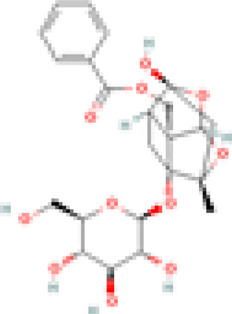	70 mg/kg or 140 mg/kg	Paeonia lactiflora Pall. [Paeoniaceae]	Kun Ming mice (outbred)	• Significantly inhibited cutaneous inflammation in mice with allergic contact dermatitis	Wang et al. (2013)
				• ↓ thymocyte proliferation in the mice with ACD.	
				• ↑ IL-4 and IL-10 production	
				• ↓ IL-2 and IL-17 levels in the serum, and in thymocyte and splenocyte culture supernatants	
				• IL-2 and IL-17 levels in the serum displayed a significant positive correlation with the severity of skin inflammation	
				• IL-4 and IL-10 levels were negatively correlated with the severity of inflammation	
Paeoniflorin 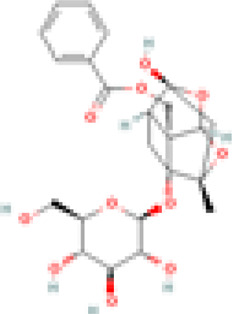	10, 20, 100, and 200 μmol/L	Paeonia lactiflora Pall. [Paeoniaceae]	Female MRL/lpr mice	• ↓ the phosphorylation of IRAK1 and its downstream proteins induced by LPS.	Ji et al. (2018)
				• Inhibited the expression of TNF-α and IL-6	
Secoiridoids - structure not available	100 mg/kg	Olea europaea L. [Oleaceae]	BALB/c mice that were 12 weeks old	Oleuropein (OL) showed antioxidant, anti-inflammatory, and immunomodulatory properties suggesting a potential application in a large number of inflammatory and reactive oxygen species (ROS)-mediated diseases	Castejon et al. (2020)
Pycnogenol (PYC) - structure not available	120 mg/day for 30 days and 60 mg/day for the following 30 days	Pinus pinaster Aiton [Pinaceae]	SLE patients	• ↓** ROS production, apoptosis, p56 (lck) specific activity, and erythrocyte sedimentation rate	Stefanescu et al. (2001)
				• ↓** SLEDAI in the treated group compared to the control group	
	25 ug/ml	Pinus pinaster Aiton [Pinaceae]	Human keratinocyte cell line, HaCaT	• Significantly inhibited IFN-gamma induced adherence of T cells to HaCaT cells	Bito et al. (2000)
				• Intercellular adhesion molecule-1 (ICAM-1) plays a major role in the IFN-gamma-induced adherence of T cells to keratinocytes	
				• Significantly inhibited IFN-gamma-induced expression of ICAM-1 expression in HaCaT cells	
				• Inhibited IFN-gamma-mediated activation of STAT1, thereby suggesting a transcriptional regulation of inducible ICAM-1 expression by PYC.	
Quercitrin 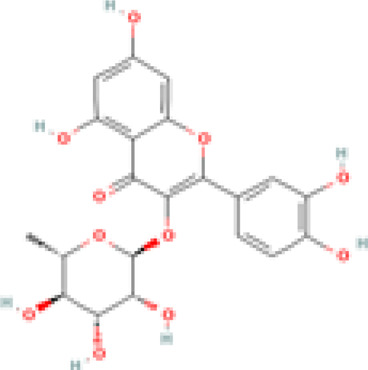	80 mg/kg	Fagopyrum tataricum (L.) Gaertn. [Polygonaceae]	Female DBA/2 and BDF1 mice, cGVHD mice	• ↓ number of serum antibodies, CD4^+^ T cell activation, as well as the expression levels of T-bet, GATA-3, and selected cytokines	Li et al. (2016)
				• ↓ expression of inflammatory genes and cytokines in the kidney, and in peritoneal macrophages	
				• Inhibited LPS-induced cytokines and the phosphorylation of ERK, p38 MAPK, and JNK in Raw264.7 cells	
				• Ameliorated the symptoms of lupus nephritis in the cGVHD mouse model possibly by the inhibition of CD4 T cell activation and anti-inflammatory effects on macrophages	
Radix Paeoniae Rubra (RPR) - paeoniflorin, albiflorin, paeonol, gallic acid, catechin and benzoic acid. (No active compound isolated - structure not available)	18 ml/kg of body weight	Paeonia officinalis L. [Paeoniaceae]	MRL/lpr mice and B6 mice	• ↓ proliferation of inflammatory cells infiltrates in the glomeruli and interstitial	Wang et al. (2020)
				• ↓ renal damage	
				• ↓ proteinuria	
				• ↓** mRNA expression levels of ICAM-1 and VCAM-1	
				• ↓** renal immunohistochemistry expressions of ICAM-1 and VCAM- 1	
				• ↓ renal mRNA expression level and the immunohistochemistry expressions of PECAM-1	
Resveratrol (Res-2) and it’s bio-enhancer piperine (RP1) 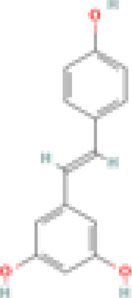 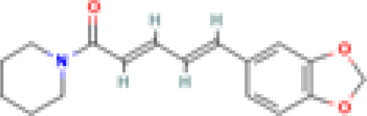	Combination of 25 mg/kg body weight of resveratrol and 2.5 mg/kg body weight of piperine or 50 mg/kg body weight of resveratrol	Piperine is extracted from Mentha × piperita L. [Lamiaceae], Resveratrol can be a plant extract however this is not specified in this study	Pristane injected BALB/c mice	• ↓ increased IFN-α, IL-6 and TNF-α	Pannu and Bhatnagar. (2020)
				• Lipogranulomas associated with injected pristane were not observed after RP-1 and high dose of resveratrol (Res-2) treatment	
				• Ameliorated lung manifestations of lupus	
				• ↓ proteinuria and creatinine in urine	
				• Regulated oxidative stress	
Prophylactic Resveratrol (P-res) and prophylactic Piperine (P-RP)	25 mg/kg of Reverastol and 2.5 mg/kg of Piperine		Pristane injected BALB/c mice	• Mitigated oxidative stress (enzyme activity of catalase, superoxide dismutase, glutathione peroxidase, and level of reduced glutathione, lipid peroxidation, and reactive oxygen species)	Pannu and Bhatnagar. (2020)
				• ↓ IL-6 by 71.60% with P-Res	
				• ↓** TNF-α by 59.70% with P-Res and 62.66% with P-RP (*p* < 0.05)	
				• ↓** creatinine level by P-RP and abrogation of proteinuria (P-Res and P-RP)	
				• P-RP ↓ immune complexes in lungs P-RP and restored histopathology of the liver	
				• P-Res extenuated lipogranulomas, histopathological manifestations in kidney, liver, and lungs, and eliminating immune complexes in liver and lungs	
				• P-RP and P-Res did not regulate auto-antibody formation	
				• P-Res ↓ the susceptibility of developing pathogenesis in murine model of lupus-like disease	
				• P-RP and P-Res combination does not prove more efficacious in preventing lupus-associated pathologies than P-Res alone	
Astilbin 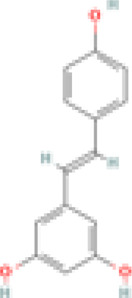 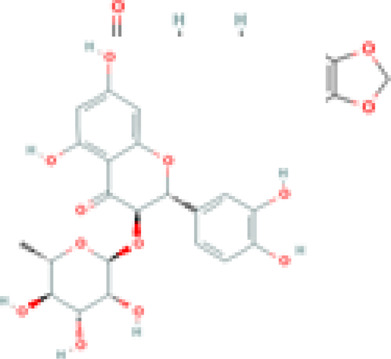	20 mg/kg every other day	Hypericum perforatum L. [Hypericaceae]	Female MRL/MpJ-Tnfrsf6lpr (MRL/lpr) mice	• ↓ splenomegaly/lymphomegaly, autoantibody production, and ameliorated lupus nephritis	Guo et al. (2015)
				• ↓** IFN-g, IL-17A, IL-1b, TNF-a and IL-6	
				• ↓ spleen CD44 hi CD62L lo activated T cells and CD138+B220- plasma cells	
				• ↓ mitochondrial membrane potential in activated T cells	
				• Downregulated expression of the co-stimulatory molecules CD80 and CD86 on LPS stimulated B cells	
Sophorae Radix (SR) (No active compound isolated - structure not available)	5.8 mg/mouse daily	Sophora flavescens Aiton [Fabaceae]	Female NZB/w F1 mice	• ↓** proteinuria	Ko et al. (2007)
				• ↓** anti-dsDNA antibodies in serum and glomerular capillaries	
				• Significant recovery from renal glomerular damage	
				• ↑** lymphocyte population	
				• ↓** interferon-gamma in splenocyte culture	
				• Findings suggest SR therapy alleviates SLE-like symptoms by correcting Th1/Th2 balance	
Tripterygium wilfordii (TW) (No active compound isolated - structure not available)	45 g per day	Tripterygium wilfordii Hook.f. [Celastraceae]	Humans (females) with SLE	• Patients receiving TW for more than 5 years had ↓** BMD levels compared with those for less than 5 years	Huang et al. (2018)
				• Prednisone induced BMD was more severe than TW-induced BMD.	
Celastrol 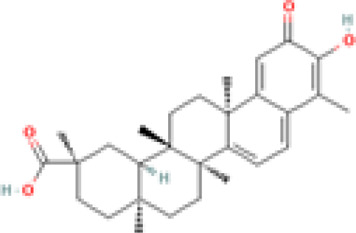	0.2 mg/kg	Tripterygium wilfordii Hook.f. [Celastraceae]	Trex1−/− mice	• Inhibits interferon regulatory factor 3 (IRF3) activation thereby down-regulation the interferon response triggered by cytosolic nucleic acids *in vitro* and *in vivo*	Liu et al. (2020)
				• Ameliorates the autoimmune phenotypes including myocarditis, aberrant interferon response, and autoantibody production, as well as the excessive T-cell activation in the Trex1−/− autoimmune disease mouse model	
Triptolide 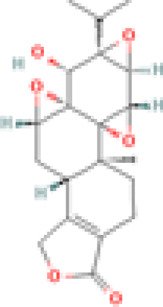	100 μg/kg per day	Tripterygium wilfordii Hook.f. [Celastraceae]	Female MRL/lpr mice	• Improved skin damage	Huang et al. (2018)
				• ↓ serum levels of IFN-γ and IL-10	
				• Improved renal histopathologic characteristics of MRL/lpr mice	
				• Downregulated the mRNA level of TLR9, TLR4, and NF-κB	
Triptolide 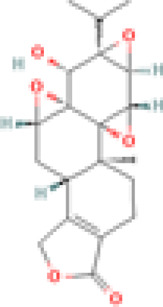	10 ng/ml	Tripterygium wilfordii Hook.f. [Celastraceae]	Healthy human PBMCs, RAW264.7 cells, B6 mouse peritoneal macrophages; C/EBPbeta^−/−^ mice, C/EBPalpha conditional KO mice		Zhang and Ma. (2010)
				• Triptolide targets CCAAT/enhancer-binding protein-alpha (C/EBPalpha), which then inhibits the transcription of the p40 promoter in inflammatory macrophages	
				• Triptolide can activate the transcription of C/EBPalpha	
				• Triptolide activates phosphorylation of Ser21 and Thr222/226 critical for C/EBPalpha inhibition of p40	
				• Kinases ERK1/2 and Akt-GSK3beta contributes to triptolide’s activation of C/EBPalpha	
D3-S1 branched polysaccharide - structure not available	30 mg·kg^−1^ day^−1^	Bupleurum smithii var. parvifolium R. H. Shan and Y. Li [Apiaceae]	Female MRL-lpr and BALB/c mice	• Elongated life span • Improved kidney function • Delayed lymphadenopathy • ↓ IFN-γ and IL-6 gene expression in the kidney	Jiang et al. (2012)
Fungus					
Ganoderma lucidum (LZ) (No active compound isolated - structure not available)	500 mg/kg/day	Ganoderma lucidum (LZ) + San Miao San (Atractylodes lancea (Thunb.) DC. [Asteraceae],Phellodendron amurense Rupr. [Rutaceae] and, Achyranthes bidentata Blume [Amaranthaceae])	MRL/lpr and BALB/c	• ↓** concentrations of anti-ds-DNA in the plasma of moderate and severe SLE.	Cai et al. (2016)
				• Gene expression levels of the induced regulatory T (iTreg) and natural Treg (nTreg) cells were ↑** than those of the Th17, Th1, and “conventional Th cells vs Treg cells” regulated genes following the LZ-SMS treatment	
				• ↑** CD4 (+), CD25 (+), Foxp3 (+), Treg cells collected from the splenic, thymic, and peripheral blood cells	
				• ↑** IL-10 (+) and Bregs collected from the splenic and thymic cells	
				• ↑** ratio of CD4 (+), CD25 (+), Foxp3 (+) and Treg cells to CD4 (+), CD25 (-) effector T cells collected from the splenic, thymic, and peripheral blood cells in LZ-SMS-treated moderate and severe SLE mice increased significantly compared with the untreated PBS group	
				• ↓** IL-21, IL-10, and IL-17A	
				• ↑** IL-2 and IL-12P70	
Bacteria					
*Lactobacillus* rhamnosus and *Lactobacillus* delbrueckiion + prednisolone	10^8^ bacteria/mice PO daily via One milliliter of MRS broth with optical density (OD) = 1.1 contained 8.8 × 10^8^ bacteria/ml. Prednisolone (5 mg/kg) in 20 μL	L. delbrueckii (DEL) subsp. lactis and L. rhamnosus	Female BALB/c mice	• Probiotics and prednisolone could delay SLE with a reduction in antinuclear antibody (ANA), anti-double-stranded DNA (anti-dsDNA), anti-ribonucleoprotein (anti-RNP), and mass of lipogranuloma	Mardani et al. (2019)
				• ↓ IFN-γ, Th1-Th17, and IL-17	
				• ↓ cytotoxic T lymphocyte (CTL) cells	
Compounds					
Macro, Nutraceutics, and Micronutrients	Unspecified	Dietary	Mice and humans	• a diet high in fiber, polyunsaturated fatty acids, vitamins, minerals, and polyphenols contain sufficient macronutrients and micronutrients can modulate inflammation and the immune system of SLE patients	Islam et al. (2020)
Arsenic trioxide 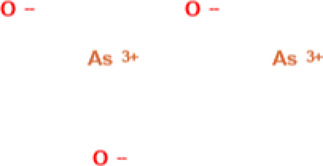	0.15 mg/kg	LUPSENIC was a phase IIa, open-label controlled, multicentre trial, evaluating dose-escalation intravenous ATO in SLE (EudraCT: 2012-002259-40; NCT: NCT01738360)	Human	• ↓ Corticosteroid dosage requirement after 24 weeks	Hamidou et al. (2021)
Taxifolin 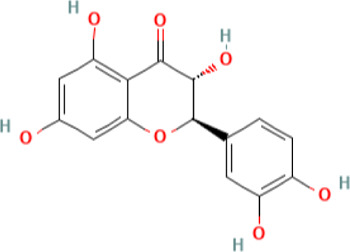	20 uM	Prepared in dimethyl sulfoxide (DMSO) at concentrations such that the final concentration of the solvent in cell suspension never exceeded 0.1%	Human cell lines (HaCaT and keratinocytes)	• Inhibited interferon gamma (IFN gamma)-induced ICAM-1 protein as well as mRNA expression in human keratinocytes	Bito et al. (2002)
				• Inhibited the activation of signal transducers and activators of transcription STAT1	
				• Inhibited the activation of protein tyrosine phosphorylation of Janus kinase (JAK)1	
				• Inhibited IFN gamma-induced ICAM-1 expression in a reconstructed human skin	
N-acetylcysteine 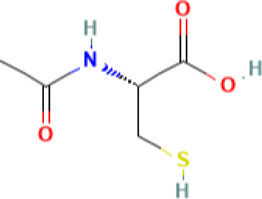	250 mg/kg/day *via* drinking water	Not specified	C57BL/6, MRL^+/+^ and MRL/lpr mice	• ↓ Rikenellaceae	Wang et al. (2021)
				• ↑ Akkeransiaceae, Erysipelotrichaceae and Muribaculaceae	
				• Attenuated the systemic autoimmunity in MRL/lpr mice	
Sodium butyrate 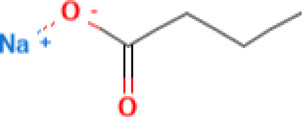	320 mg/kg, 0.1 ml/10 g body weight three times per weekly	Sodium butyrate (Sigma-Aldrich, United States)	Female MRL/lpr mice and BALB/c wild-type mice	• ↑** abundance of Firmicutes, Clostridia, Clostridiales, Lachnospiraceae, Ruminococcaceae, Peptostreptococcaceae, Ruminiclostridium, Oscillibacter, Romboutsia, Lachnoclostridium, Coprococcus, Ruminococcus, Clostridium leptum, and Dorea_spp	He et al. (2020)
				• ↓** in proportion of Bacteroidetes, Bacteroidia, and Bacteroidales	
				• Butyrate supplementation ameliorated gut microbiota dysbiosis characteristic of SLE.	
Tetraarsenic tetrasulfide 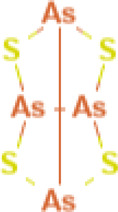	1,000 mg/g body weight by oral	Not specified	BXSB mice	• Significantly improved monocytosis in spleen	Zhao et al. (2013)
				• ↓** serum IL-6	
				• Ameliorated skin, liver, and renal disease with mild side effects	
				• Suppressed immune complex deposition, mesangial proliferation and inflammatory cell infiltration in kidney and liver	
Ethyl pyruvate 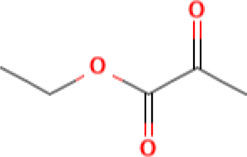	100 mg/kg	Not specified	Female SLE patients between 13 and 43 years of age bone marrow supernatant and female MRL/lpr mice	• Alleviated the clinical aspects of lupus nephritis	Ji et al. (2019)
				• ↑ survival of MRL/lpr mice	
				• Reversed the senescent phenotype of bone marrow mesenchymal stem cells from MRL/lpr mice	
Herbal formula					
Specific active chemical compounds was not mentioned in article	1.2 g/kg PO daily	Sairei-to = 12 herbal ingredients: bupleuri radix, pinelliae tuber, scutellariae radix, ginseng radix, glycyrrhizae radix, zizyphi fructur, zingiberis rhizoma, atractylodis lanceae rhizoma, polypourus, alismatis rhizoma, hoelen and cinnamoni cortex	MRL/lpr mice	↓ IgG deposition at the dermo-epidermal junction, titers of anti-DNA antibodies and rheumatoid factor, and lymphoproliferation	Kanauchi et al. (1994)
Specific active chemical compounds was not mentioned in article	250 mg/kg PO daily	Longdan Xiegan Tang (LXT) = 10 ingredients: Gentiana rigescens Franch, Scutellaria baicalensis Georgi, Gardenia jasminoides	MRL/lpr mice	• ↑ splenic CD3^+^CD4^+^, CD3^+^CD8^+^, and CD4^+^CD25^+^ T cells	Lee and Chang. (2010)
		Ellis, Alisma orientalis (Sam.) Juzep, Clematis Montana Buch.-Ham., Plantago asiatica L, Angelica sinensis (Oliv.) Diels, Rehmannia glutinosa Libosch, Bupleurum chinense DC, and Glycyrrhizae uralensis Fisch		• ↓ IFN-gamma, TNF-alpha, anti-dsDNA antibody	
				• ↓ IgG immune complex deposits in the glomeruli	
				• Restored kidney glutathione levels, thereby limiting the toxic effects of the inflammatory mediators iNOS and COX-2, which are overproduced in MRL/lpr mice	
				• Up-regulated phosphoglycerate kinase 1	
				• Down-regulated ferritin light chain 1, selenium-binding protein 2, and alpha-enolase	
				• ↓ oxidative stress associated with disease progression in MRL/lpr mice	
Loganin and paeoniflorin 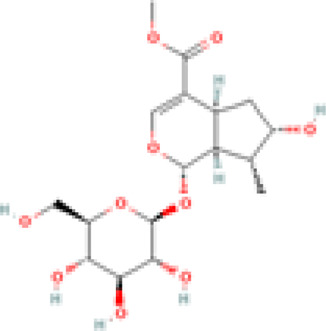 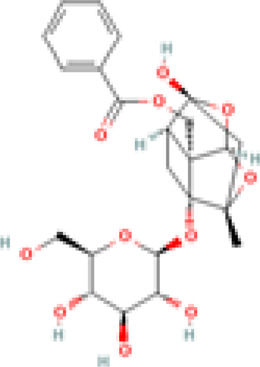	Sachet of granules (10 g) in 200 ml of hot water; orally Twice daily for 12 weeks	Zi Shen Qing (ZSQ) = Radixastragali, Rehmannia glutinosa libosch, Fructus Corni, Paeonia lactiflora, Herba Hedyotis Diffusae, and Cortex Moutan Radicis	SLE patients with SLEDAI of 5–14	• ↓** SLEDAI-2000	Zhong et al. (2013)
				• Improved the withdrawal dosage of corticosteroids and the incidence of disease flare-up	
Specific active chemical compounds was not mentioned in article	2.7 g of LWDHW and 125 mg of Dan-Chi San three times daily	Dan-Chi-Liu-Wei combination (DCLWC) + prednisolone	SLE patients with SlEDAI of 2–12	• ↓ SLEDAI score in the experimental group	Liao et al. (2011)
				• Adding-on DCLWC to conventional therapy for the treatment of SLE was safe and might have a borderline effect in decreasing disease activity, but it was not possible to taper the dosage of steroid after 6 months of clinical trial	
Paeoniflorin, catalpol, ferulic acid, liquiritin, rutin, hesperidin, quercetin, asiaticoside, glycyrrhizic acid (mostly contains paeoniflorin)	Intragastric administration with 2 ml/100 g	Jieduquyuziyin prescription (JP)	Male Sprague Dawley (SD) rats and female MRL/lpr lupus mice	• ↓ phosphorylation of IRAK1 and its downstream proteins induced by LPS	Ji et al. (2019)
				• Inhibited the expression of TNF-α and IL-6	
				• JP may inhibit the activation of peritoneal macrophages in MRL/lpr mice by downregulating the IRAK1-NF-κB signaling pathway, and IRAK1 may be a potential target for JP treatment of SLE.	
	18 ml/kg body weight per day	Jieduquyuziyin prescription (JP)	Mice (MRL/lpr and B6)	• ↓** percentages of Th17, IL-17, and RORγt	Shui et al. (2015)
				• Inhibited CaMK4 expression	
Toxins					
Specific active chemical compounds was not mentioned in article	40 ug/kg	Naja Naja Atra Venom (NNAV) = 3 ingredients: cobra venom factor, cardiotoxins, and neurotoxins	MRL/lpr mice	• Protected against including skin erythema and proteinuria	Zhu et al. (2014)
				• ↓ levels of glutamate pyruvate transaminase and creatine kinase	
				• ↑ serum C3	
				• ↓ concentrations of circulating globulin, anti-dsDNA antibody, and inflammatory cytokines IL-6 and TNF-α	
				• ↓ lymphadenopathy and renal injury	

### 2.4 Methodological Quality Assessment

We assessed the risk of bias using the ROBINS-I tool (Sterne et al., 2016). Our quality assessment template included the following metrics: 1) Bias in selection of participants: Was the selection of participants into the study (or into the analysis) based on participant characteristics observed after the start of intervention?; 2) Bias due to deviations from intended interventions: What is the predicted direction of bias due to deviations from the intended interventions?; 3) Bias due to missing data: What is the predicted direction of bias due to missing data?; 4) Bias in measurement of outcomes: What is the predicted direction of bias due to measurement of outcomes?; 5) Bias in selection of the reported result: What is the predicted direction of bias due to selection of the reported result?; 6) Bias in the classification of interventions: What is the predicted direction of bias due to classification of interventions?; 7) Risk of bias due to confounding: is there potential for confounding of the effect of the intervention in this study?; 8) Overall bias - the risk of bias judgment: What is the overall predicted direction of bias for this outcome? (Refer to Supplementary Table S2).

### 2.5 Data Extraction

Our data extraction template included the following entries for reviewers to complete: 1) study ID; 2) title; 3) country in which the study was conducted; 4) notes; 5) aim or objective of the study; 6) study design (e.g., randomized clinical trial, case report, etc.); 7) publication date; 8) study funding sources; 9) possible conflicts of interests for study authors; (10) participants - population description (mice, humans, cell lines, etc.); 11) inclusion criteria; 12) exclusion criteria; 13) the total number of participants; 14) total number of experimental repeats; 15) plants/compounds/extracts tested; 16) active ingredients tested (if known); 17) controls used (e.g., vehicle, positive controls, negative controls, etc.); 18) primary outcome - cutaneous findings - Was the treatment efficacious (yes/no)? Statistically significant (yes/no)? 19) primary outcome - inflammation - Was the treatment efficacious (yes/no)? Statistically significant (yes/no)? (20) Primary outcome - other organ systems (e.g., kidney, brain, etc.).

Was the treatment efficacious (yes/no)? Statistically significant (yes/no)?

## 3 Results and Context of Included Studies

### 3.1 Characteristics of Included Studies

**FIGURE 2:**
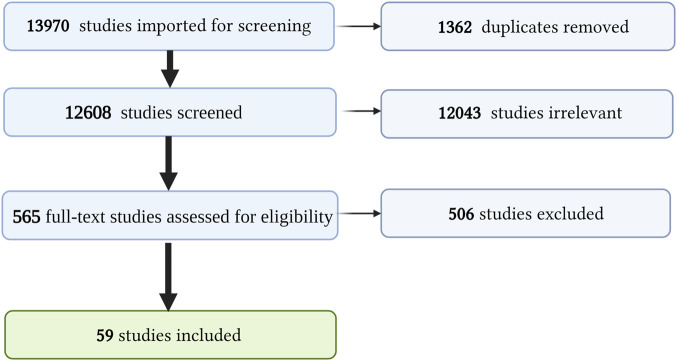
PRISMA diagram. Summary of our systematic review process and screening. Created with BioRender.com.

Our search was conducted as outlined in the *Methods* and Supplementary Full Ovid Medline Search Strategy. This yielded 13,970 studies, of which 1,362 were duplicates. We screened 12,608 abstracts, of which 12,043 were irrelevant, leaving 565 full-text studies we assessed for eligibility. Of these, 506 were excluded for the following reasons: 220 were not pertinent to Cutaneous Lupus, 132 addressed a natural compound but not pertinent to cutaneous lupus, 62 full texts not in English, 37 did not address specific natural compounds and plant extracts, 41 full articles and references not available, 11 HCQ, 4 discussed natural compound that causes SLE. A total of 59 studies were included for data extraction. A PRISMA flow diagram outlining our systematic review process is presented in Figure 2.

### 3.2 Consensus Risk of Included Studies

The inter-rater reliability, the extent of agreement among data collectors, was assessed using Cohen’s kappa. Cohen’s Kappa, a metric often used to assess the agreement between two raters, was performed during the abstract/title screening and the full-text review (McHugh, 2012). The review of the title/abstract screening conducted by JEL and ASJ resulted in a Cohen’s Kappa of 0.539, indicating moderate agreement (McHugh, 2012). The reviews conducted by JEL and JMR together resulted in a Cohen’s Kappa of 0.454, indicating moderate agreement (McHugh, 2012). In the full-text review, article assessments conducted by JEL and ASJ together produced a Cohen’s Kappa of 0.574. This suggests a moderate agreement between these two reviewers (McHugh, 2012). We assessed the full texts for extraction for low, medium, and high confidence studies. The consensus risk of studies was evaluated using the quality assessment. The overall risk of bias for these studies is presented in Supplementary Table S2, including information about qualitative vs quantitative studies, in column “Overall bias - risk of bias judgment” and column “Overall bias - risk of bias judgment supporting text”.

### 3.3 Synthesized Findings

**FIGURE 3:**
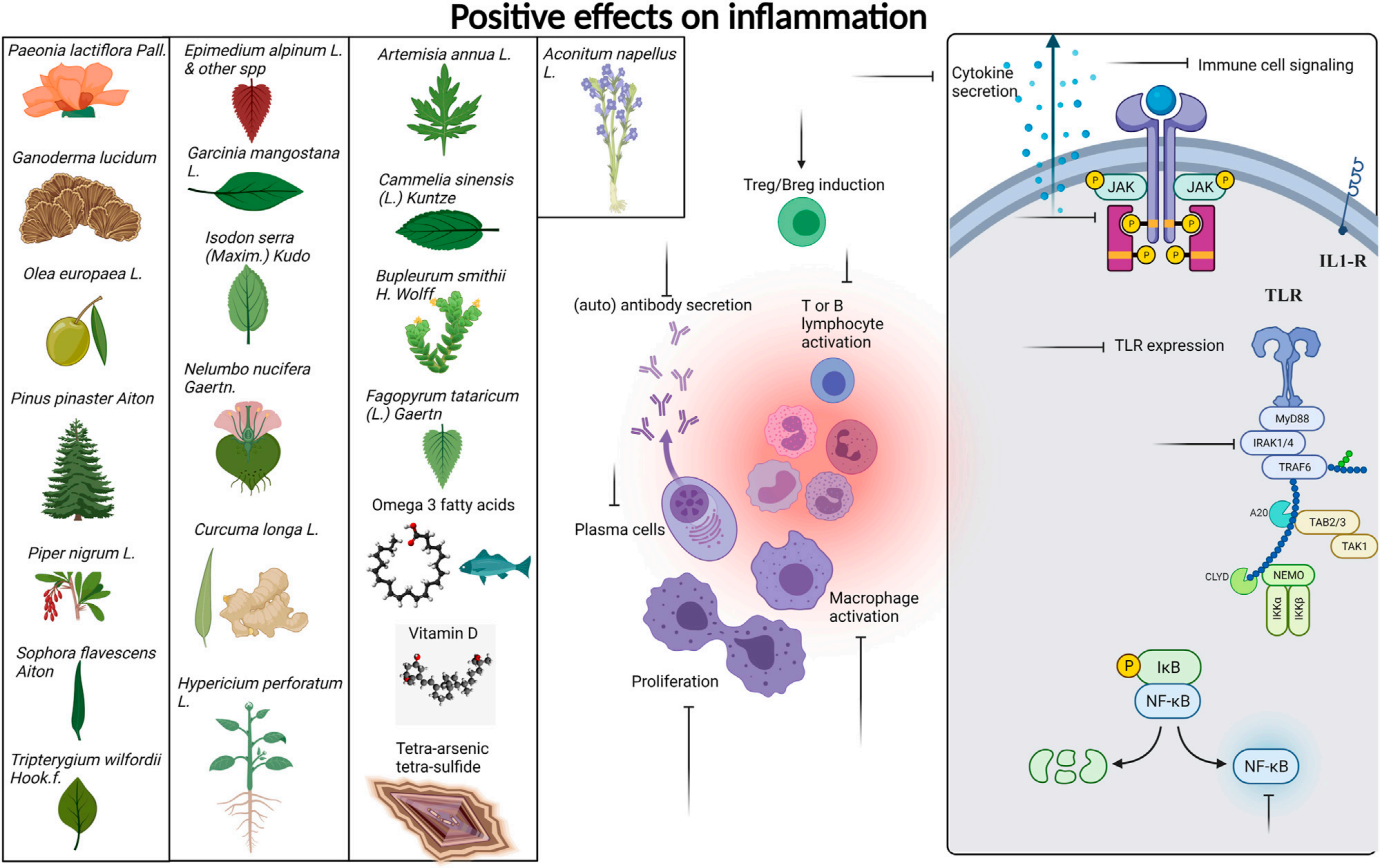
Compounds with positive effects on inflammation. Plant extracts and natural compounds identified in our search that had positive effects on inflammation are presented at left. Specific pathways of immune cells targeted by these extracts and compounds are presented at right. Inflammatory processes targeted by natural compounds include generation of Tregs and Bregs, inhibition of lymphocyte activation, proliferation, cytokine signaling, and secretion, reduced TLR expression, and inhibition of JAK/STAT NFkB and IRAK signaling pathways. A detailed list of which compounds provided which effects are provided in Table 1. Created with BioRender.com.

**FIGURE 4:**
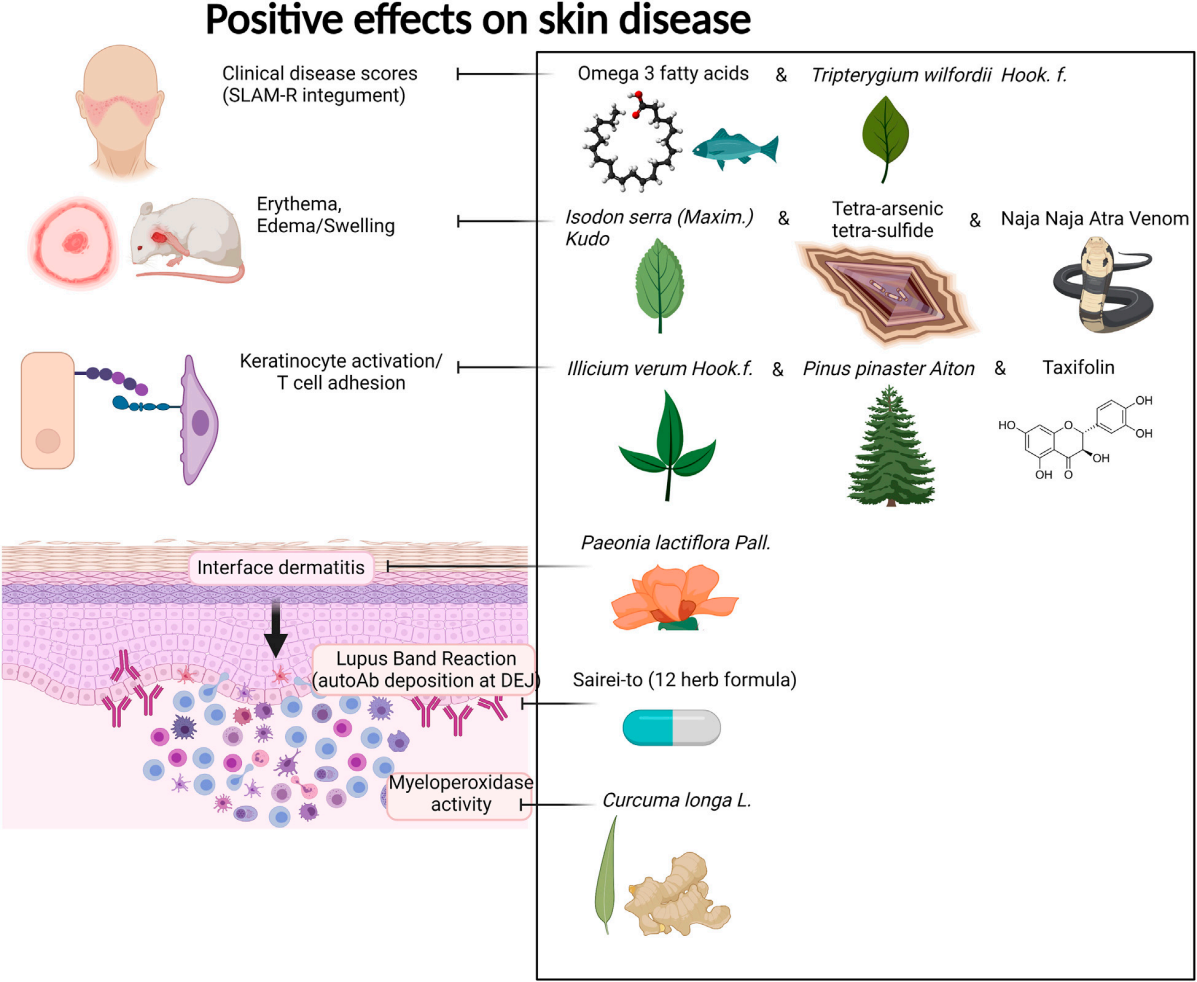
Compounds with positive effects on skin disease. Omega 3 fatty acids and *Tripterygium wilfordii Hook. f.* demonstrated positive effects on clinical skin disease in CLE and SLE patients. *Isodon serra (Maxim.) Kudô*, tetra-arsenic tetra-sulfide, and Naja Naja Atra Venom exhibited positive effects on erythema and edema in mouse models of lupus. *Illicium verum Hook. f., Pinus pinaster Aiton*, and the active compound taxifolin, which can be isolated from these plants, demonstrated positive effects on keratinocyte activation and T cell adhesion in *in vitro* and *in vivo* mouse assays. *Paeonia lactiflora Pall.* demonstrated positive effects on interface dermatitis, and Sairei-to (12-herb formula) prevented lupus band reaction. Last, *Curcuma longa L.* (curcumin, turmeric) reduced myeloperoxidase activity of granulocytes in the skin. Created with BioRender.com.

We provide Table 1 demonstrating our 59 included studies, breaking down the results by both category and biological effect. This includes information about what these compounds were tested in (people, mice, cells) and the risk of bias as assessed by ROBINS-I in Supplementary Table S2. Below we provide a detailed description of the findings for each compound/family and the effects exerted on skin inflammation or inflammation. We also provide Figure 3 summarizing natural compounds and extracts identified in this systematic review that had positive effects on inflammation and those that had positive effects on skin disease in Figure 4, including a summary of the molecular pathways targeted by these compounds.

#### 3.3.1 Fish Oils

Multiple human studies utilizing oil extracts have shown promising application to CLE. A randomized control trial, which assessed the effect of dietary supplementation with omega-3 fish oils with or without copper on disease activity in systemic lupus erythematosus (SLE), has improved skin findings among the studied patients (Duffy et al., 2004). Specifically, the components of the Systemic Lupus Activity Measure index (SLAM-R) index most affected by fish oil supplementation were the integument, neuromotor, and laboratory domains (Duffy et al., 2004). Additionally, a randomized control trial that observed the clinical effect of dietary supplementation with low-dose w-3-polyunsaturated fatty acids on SLE disease activity reported similar findings with additional disease activity assessments (Wright et al., 2008). There was also a significant reduction in the British Isles Lupus Assessment Group index scores (BILAG) in the general symptoms, cutaneous, musculoskeletal, cardiorespiratory, and vasculitis scores (Wright et al., 2008). In patients receiving fish oil, there was a significant reduction in SLAM-R at 12 weeks and 24 weeks of intervention (Wright et al., 2008). When using the SLAM-R index, there was a marked reduction in the individual scores at 12 weeks for constitutional symptoms and joints, and at 24 weeks, there was a notable reduction in constitutional symptoms, integument, neuromotor and joint scores (Wright et al., 2008). A smaller randomized control trial aimed to assess the efficacy of Seluang fish oil against proinflammatory cytokines, vitamin D levels, and clinical conditions of SLE (Partan et al., 2019). Seluang fish oil was clinically efficacious via the Mexican Systemic Lupus Erythematosus Disease Activity Index (MEX SLEDAI) score compared to placebo (Partan et al., 2019). However, other data suggests that change in Safety of Estrogens in Lupus Erythematosus National Assessment (SELENA) did not indicate a significant difference between fish oil treatment and controls (Arriens et al., 2015).

Studies on fish oil supplementation have supported clinical and overall health improvement. When comparing score changes for the energy/fatigue and emotional well-being subscale of the Rand 36-Item Short Form Health Survey (Rand SF-36), there was a trend in improvement for the fish oil group compared to the placebo group (Arriens et al., 2015). Additional data notes that subjective clinical and outcome improvement in all patients receiving fish oil and/or copper compared to placebo (Duffy et al., 2004). Other data shows that fish oil patients improved global disease activity compared to the placebo patients based on the Physical global assessment (Arriens et al., 2015).

These oils can also reduce inflammation in SLE patients, warranting investigation into whether they can be repurposed for CLE. Disease-related inflammatory markers such as ESR have also been shown to be affected by fish oil. Some studies have observed a considerable reduction in the fish oil group compared to the placebo group and placebo (Arriens et al., 2015). Among the cytokines, chemokines, and growth factors, fish oil-treated SLE patients increased IL-13 levels and decreased IL-12 levels (Arriens et al., 2015). Seluang fish oil was specifically able to increase serum vitamin D levels compared with placebo. Seluang fish oil treated lupus patients presented with decreased IL-1, IL-6, and IL-17 levels compared to the placebo group (Partan et al., 2019). In an *in vitro* study performed on human peripheral blood mononuclear cells (PBMCs) of lupus patients, there is evidence that the phenolic fraction from extra virgin olive oil also has anti-inflammatory properties. The phenolic fraction in cell cultures significantly reduced IL-6, TNF-alpha, IL-10 (Aparicio-Soto et al., 2017). This substance also significantly prevented induced IkappaBalpha degradation and inhibited ERK phosphorylation in healthy donors and lupus patients (Aparicio-Soto et al., 2017).

#### 3.3.2 Vitamins

Numerous studies have tested the level of Vitamin D (cholecalciferol) in CLE patients since many of them experience photosensitivity and therefore practice sun avoidance. Anti-inflammatory properties of vitamin D are thought to be important in autoimmune diseases like Multiple Sclerosis and SLE. A cross-sectional study assessed the association of serum vitamin D levels with CLE disease activity, which revealed that the presence of CLE raised the odds of having vitamin D deficiency (Cutillas-Marco et al., 2014). Age and disease duration were also associated with higher odds of vitamin D deficiency (Cutillas-Marco et al., 2014). The results of a comparative study found that virtually all CLE patients who strictly avoid sun exposure and wear UV-blocking sunscreen to avoid CLE exacerbations suffer from vitamin D deficiency all year round (Heine et al., 2010; Cutillas-Marco et al., 2014). A cohort study details similar findings, noting that vitamin D levels were significantly lower among sun avoiders and daily sunscreen users (Cusack et al., 2008). Significantly higher vitamin D levels were found among those who took cholecalciferol (vitamin D3) supplements (Cusack et al., 2008). Research comparing populations of different skin types and CLE disease status found that skin type also had a significant effect on 25-OH vitamin D levels when controlling for disease status (Word et al., 2012). African Americans had significantly lower levels of 25-OH vitamin D than Caucasians and Hispanics when controlled for CLE disease status and season (Word et al., 2012). Studying the prevalence and risk factors for vitamin D deficiency among Asian patients living in high UV exposure areas, there was a significant positive correlation between hours of sunlight exposure per day and vitamin D levels for the controls but not for the CLE patients (Grönhagen et al., 2016).

Vitamin D supplementation may improve CLE disease severity in some cases, but there is also some evidence to the contrary. A prospective observational study, which aimed to assess the effect of vitamin D supplementation on CLE disease activity, showed clinical improvement in CLE patients taking vitamin D supplementation (Cutillas-Marco et al., 2014). Their data showed significant clinical improvement after one year in the treatment group (Cutillas-Marco et al., 2014) 2014). Cutaneous Lupus Erythematosus Disease Area and Severity Index Activity Score (CLASI A) decreased from 2.7 ± 2.9 to 0.9 ± 1.4 (*p* = 0.003); however, the Cutaneous Lupus Erythematosus Disease Area and Severity Index Damage Score (CLASI D) did not significantly change in this study population (Cutillas-Marco et al., 2014). In spite of this, there was a trend towards fewer exacerbations per year in the treatment group (Cutillas-Marco et al., 2014). Other data has shown that vitamin D may not contribute to improvement in disease severity. A cohort study did not find a significant inverse correlation between change in the Systemic Lupus Erythematosus Disease Activity Index 2000 (SLEDAI-2K) and changes in vitamin D level over a period of 12 months (Yap et al., 2015). Similarly, a cross-sectional study found no correlation between CLASI activity scores and 25-OH vitamin D levels in CLE African American or Caucasian/Hispanic subjects (Word et al., 2012). Additionally, the mean serum vitamin D levels did not differ significantly between those with CLE who took vitamin D supplementation and those who did not (Grönhagen et al., 2016). Other findings on vitamin D and lupus provide evidence on the anti-inflammatory potential of vitamin D in SLE patients; an *in vitro* and *ex vitro* study was conducted in which vitamin D treatment was administered to SLE peripheral blood mononuclear cells (PBMC). This study found that Vitamin D downregulated TLR3, decreased the relative expression levels of TLR7 mRNA, and decreased the expression of TLR9 in SLE PBMC compared to healthy controls (Yazdanpanah et al., 2017). Thus, the clinical effects of vitamin D supplementation are controversial, though *ex vivo* studies point to potential positive effects on disease parameters.

#### 3.3.3 Plant Extracts

##### Artemisia annua L. [Asteraceae]

*Artemisia annua L. [*Asteraceae*]* has widely been used to treat rheumatic autoimmune diseases such as lupus erythematosus and rheumatoid arthritis in China and is known for its antioxidant characteristics and high nutritional value in amino acids and vitamins. Other pharmacological activities of this plant include immunosuppression and anti-inflammation (Das, 2012). These characteristics, which form the basis of treatment, were demonstrated in the immune and pro-inflammatory pathways of most reviewed articles in this study—however, the cutaneous effects of the plant were not explicitly stated.

In a controlled *in vitro* mouse study, Musallam et al. evaluated the immunosuppressive potential of an ethanol extract of Artemisia annua on mouse splenocyte proliferation (Musallam et al., 2009). In addition, the authors sought to identify specific antibodies and cellular immune responses in the ovalbumin (OVA) immunized mice (Musallam et al., 2009). EEAA significantly reduced Concanavalin A (Con A) and lipopolysaccharide (LPS)-stimulated splenocyte proliferation *in vitro*. Furthermore, the study provides evidence that EEAA reduces the levels of OVA-specific serum IgG, IgG1 and IgG2b antibodies in mice that have been immunized with OVA (Musallam et al., 2009). Although the researchers did not use vehicle controls in this study, they did include both positive controls and objective readouts.

In an *in vivo* study by Wu et al., the lifespan of murine lupus models and systemic features such as lymphadenopathy improved when treated with the artemisinin analog SM934 (Wu et al., 2016). SM934 significantly reduced serum levels of anti-nuclear antibodies (ANAs), along with interleukins 6, 10, and 21 (IL-6, IL-10, and IL-21) (Wu et al., 2016). This study also found that SM934 increased the number of quiescent B cell numbers and effectively restored the B-cell compartment in the spleen of MRL/lpr mice. Additionally, the number of activated B cells was reduced in the experimental group (Wu et al., 2016). B cells, T cells, and cytotoxic T cells dysregulation in CLE pathogenesis have been established by several studies (Garelli et al., 2020), and recent gene expression studies of canine CLE by our group revealed a high cell-type score for B cells, T cells, and cytotoxic T cells (Garelli et al., 2021) (Amudzi et al. in press), indicating a potential use of artemisinin derivatives for veterinary medicine.

Wu et al. also examined the effect of artemisinin derivatives on human peripheral blood mononuclear cells (PBMCs). The production of plasma cells (PC) was hindered, whereas SM934 triggered activation and proliferation of B cells and antibody secretion *ex vivo* (Wu et al., 2016). Furthermore, SM934 downregulated Toll-like receptor (TLR) 7 and 9 mRNA expression, MyD88 protein expression, and NFκB phosphorylation, thereby interfering with the B-cell intrinsic signaling pathways (Wu et al., 2016)*.* We posit that topical formulations of artemisia species may elicit a similar response based on these results, thereby reducing inflammation and autoimmunity and eventually improving cosmesis and quality of life for CLE patients.

##### Bupleurum smithii var. parvifolium R. H.Shan and Y. Li [Apiaceae]

*Bupleurum smithii var. parvifolium R. H. Shan and Y. Li [*Apiaceae*]* (BP) is a member of a large plant genus with 150 known species (Neves and Watson, 2004). Evidence from previous studies shows BP’s immunomodulatory function on macrophages (Jiang et al., 2012). In this study, BP extract was effective in lengthening the life span of MRL/lpr mice in a non-randomized experimental study (Jiang et al., 2012). This outcome was a function of reduced autoantibodies, improved kidney function, and delayed lymphadenopathy in the mice models (Jiang et al., 2012). Furthermore, BP extract demonstrated an inhibitory effect on the complement and macrophages activation and suppression of interferon-gamma (IFN-γ) and IL- 6 gene expression in the kidney (Jiang et al., 2012). Considering that both SLE and CLE are interferon-dependent disorders, BP may be effective for both conditions.

##### Camellia sinensis (L.) Kuntze [Theaceae]

*Camellia sinensis (L.) Kuntze [*Theaceae*]*, commonly known as green tea, has been widely studied for beneficial health effects. Many of these beneficial health effects have been ascribed to one of the most potent compounds called epigallocatechin-3-gallate (EGCG), which is fairly safe in animals and humans. Epigallocatechin-3-gallate (EGCG) is a JAK inhibitor (Tedeschi et al., 2002; Ning et al., 2015; Hamed et al., 2018) and a potent antioxidant (Tsai et al., 2011) effective in various illnesses, including autoimmune diseases. Our search yielded a study by Otton et al., who found that green tea extract could reduce the expression of miR-335 in adipose tissue in response to TNF (Otton et al., 2018). The anti-inflammatory properties of EGCG on adipose tissue in obese mice were observed while examining miR-335 changes. The results from their study suggest an increased energy expenditure of high-fat diet-fed mice, decreased weight gain, consequently resulting in a reversal of metabolic complications and attenuation of inflammation.

Since metabolic complications contribute to increased morbidity and mortality in SLE patients, as mentioned in a study by Szabo et al., where 30% of SLE patients at the time of diagnosis were found to have dyslipidemia with elevations in total cholesterol (TC), low-density lipoprotein (LDL), triglyceride (TG), and apolipoprotein B (ApoB) (Szabó et al., 2017), safe and efficacious compounds that mitigate the metabolic complications of lupus erythematosus will be ideal. In an *in vitro* study, EGCG was found to inhibit fibroblast growth and collagen production in a keloid model (Park et al., 2008).

Additionally, EGCG has been found to increase regulatory T cells that exert a crucial role in immune function, modulate cytokine production, and suppress autoimmune disease (Wu et al., 2021). Given that high TNF expression in CLE patients predicts poor response to hydroxychloroquine (Zeidi et al., 2019), it is possible that EGCG could provide an additive benefit for patients currently treated with hydroxychloroquine. A further investigation is necessary to determine which JAK/STAT pathway primarily drives the CLE response. In the future, green tea extract may serve as a promising treatment for CLE.

##### Curcuma longa L. [Zingiberaceae]

Turmeric, scientifically known as *Curcuma longa L. [*Zingiberaceae*]*, is a medicinal spice widely known for its anti-inflammatory and antioxidant effects. The active ingredient, curcumin (diferuloylmethane), has a broad range of bioactive compounds that are safe and effective against various diseases, including autoimmune diseases. From our searches, curcumin shows several mechanisms of action that could be beneficial for CLE. Zhao et al. demonstrated that curcumin reduces proteinuria, renal inflammation, serum anti-dsDNA antibodies, splenomegaly, and NLRP3 inflammasome activation in MRL/lpr mice *in vitro* and *in vivo* assays (Zhao et al., 2019). While it was not explicitly studied, the authors acknowledged curcumin’s potential use for cutaneous lesions.

Kurien et al. demonstrated a significant reduction in binding of autoantibodies to their cognate antigens by turmeric up to 70% in SLE patients, though this inhibition was not specific to autoimmunity (Kurien et al., 2010). In their study, the authors also examined preparations of curcumin and turmeric to enhance bioavailability. They also observed a 12-fold increase in the solubility of curcumin and a 3-fold increase in the solubility of turmeric by applying heat to them in water for 10 min. However, there was a 43% inhibition of Ro60 antigen-antibody binding by heat-solubilized curcumin compared with a 65% inhibition with heat-solubilized turmeric using sera from SLE patients, suggesting that higher inhibition can be achieved with turmeric extract than with purified curcumin. Since a significant setback of turmeric or curcumin’s full pharmacologic effects in experimental studies is their inability to dissolve in an aqueous medium, heated turmeric, which maintains the spices’ safety and efficacy, might be a better therapeutic approach when considering topical formulations for CLE. In addition, heat-solubilized Tumeric was found to bind to a wide range of protein receptors (Ro273 MAP), suggesting its ability to affect multiple signaling pathways, including cytokines and chemokines: type 1 interferons, CXCL10, JAK-STAT, and PRR signaling (Kurien et al., 2010; Wenzel, 2019). Though anti-SSA/Ro60 has been independently associated with SLE compared with Sjögren’s syndrome (SS) and other systemic autoimmune diseases, it is more frequently specific for CLE (Menéndez et al., 2013).

Castangia et al. examined curcumin, and quercetin-loaded nanovesicles and their effects counteracting phorbol ester 12-O-tetradecanoylphorbol-13-acetate (TPA) induced skin inflammation in a mouse model both *in vitro* and *in vivo* (Castangia et al., 2014). Quercetin liposomes (59%) and curcumin liposomes and polyethylene glycol (PEG)-PEVs (∼68%) inhibited myeloperoxidase activity. PEG-PEVs provided an extensive re-epithelization of TPA-damaged skin. Quercetin is further discussed in the next section.

##### Fagopyrum tataricum (L.) Gaertn. [Polygonaceae]

Quercetin is a plant polyphenol with many biological properties that include anti-inflammatory, antiviral, antiplatelet aggregation, and capillary permeability *in vitro* and in animals (Li Y. et al., 2016). This study demonstrates that quercetin derived from Tartary buckwheat (*Fagopyrum tataricum (L.) Gaertn. [*Polygonaceae*]*) can reduce the number of serum antibodies, CD4^+^ T cell activation, and the expression levels of T-bet, GATA-3, and selected cytokines in female DBA/2 mice with SLE-like presentation (Li W. et al., 2016). The proposed mechanism of action involves the inhibition of CD4 T cell activation and anti-inflammatory effects on macrophages (Li W. et al., 2016). This ability of quercetin to inhibit CD4 T DNA demethylation in SLE and SCLE CD4 (+) T cells contributes to overexpression of the cytotoxic effector molecule perforin (Luo et al., 2009). Considering the inhibitory activity on CD4 T cells, LPS-induced cytokines, and the phosphorylation of ERK, p38 MAPK, and JNK in Raw264.7 cells (Li W. et al., 2016), quercetin proves to be a potent immunomodulator that can be useful in targeting CLE. However, the bioavailability of quercetin in humans is low (0–50%) with a half-life of 1–2 h (Graefe et al., 1999), warranting further development of derivatives and second-generation compounds.

##### Epimedium alpinum L. [Berberidaceae]

Icaritin (ICT), extracted from *Epimedium alpinum L. [Berberidaceae]* and other members of this family, has been used in TCM for a long time. Liao et al. examined the effect of ICT on human SLE peripheral blood mononuclear cells (PBMC) and MRL/lpr mice and found that this extract inhibited CD4 (+) T cell overactivity, promoted FoxP3-IL17a balance, and enhanced Treg cell suppression (Liao et al., 2016). The observed Foxp3/IL17a balance in ICT treatment was postulated to be a factor of the increase in STAT5b expression and through histone methylation modification (Liao et al., 2016). ICT’s effect on IL-17 suggests it may have a far-reaching effect on lupus immunomodulation because IL-17 is upregulated in SCLE patients and increases inflammatory cytokines and chemokines, neutrophil recruitment, stimulates T cells, and increases the production of autoantibodies (Méndez-Flores et al., 2016). *In vivo*, ICT conferred immunosuppressive action in MRL/lpr mice by reducing Th1/Th2 cytokines, and by interfering with the activation of T cells by increasing NF-AT luciferase activity in Jurkat-NF-AT-luc T cells (Li et al., 2012). Lastly, considering that Th1-biased inflammatory immune response can recruit CXCR3-expressing T-lymphocytes to the skin (Wenzel et al., 2005), ICT action on Th1 shows its potential to be beneficial in CLE therapy.

##### Garcinia mangostana L. [Clusiaceae]

In this study, Li et al. demonstrated the medicinal potential of isogarcinol extract from *Garcinia mangostana L. [Clusiaceae].* Treatment of DBA/2 mice resulted in significantly reduced proteinuria, decreased the number of serum antibodies, and lowered the renal histopathology score (Li et al., 2015). Isogarcinol also decreased the expression of inflammatory genes and cytokines in the kidneys and peritoneal macrophages and alleviated the abnormal activation of CD4 T cells (Li et al., 2015). It was determined that Isogarcinol confers a significant immunosuppressive effect on SLE (Chen et al., 2017), and works by regulating abnormal activation of CD4 T cells, which cause inflammation (Li et al., 2015). A different study demonstrated that Isogarcinal prevented abnormal T cell distribution and inhibited CD4+T cell differentiation into Th17 cells in mouse spleens (Chen et al., 2017). Treatment with isogarcinol significantly inhibited aberrant proliferation and differentiation of keratinocytes and inhibition of the expression of genes involved in the IL-23/Th17 axis, TNF-α, IL-2, and IFN-γ in the skin of mice (Chen et al., 2017). Furthermore, Chen et al. found that isogarcinol strongly inhibited inflammatory factor expression in lipopolysaccharide (LPS)-stimulated HaCaT cells (Chen et al., 2017). These combined findings provide evidence of the immunomodulatory activity of isogarcinol systemically and specifically on the skin, thereby proving the potential application of this extract in CLE treatment.

##### Hypericum perforatum L. [Hypericaceae]

Astiblin from *Hypericum perforatum L. [Hypericaceae]* (St. John’s wort) proved effective in significantly reducing the serum level of IFN-g, IL-17A, IL-1b, TNF-a, and IL-6 in lupus-prone mice (Guo et al., 2015). This study shows that the extract induced the development of splenic CD44 hi CD62L lo T cells and subsequently reduced CD138+B220- plasma cells (Guo et al., 2015). Additionally, IVE treatment decreased mitochondrial membrane potential in activated T cells and decreased expression of the co-stimulatory molecules CD80 and CD86 in LPS-stimulated B cells (Guo et al., 2015). According to these results, astilbin inhibits disease development in lupus-prone mice by reducing the functionality of activated T and B cells (Guo et al., 2015). This mechanism is particularly relevant to CLE, which requires both T and B lymphocytes (Prinz et al., 1996; Kansal et al., 2019; Mougiakakos et al., 2021).

##### Illicium verum Hook.f. [Schisandraceae]

This study investigated the anti-inflammatory effects and regulatory mechanisms of *Illicium verum Hook. f. [Schisandraceae]* extract (IVE) in the human keratinocyte cell line HaCaT. IVE significantly inhibited IFN-γ-induced mRNA and protein expression of ICAM-1 in HaCaT cells (Sung and Kim, 2013). IVE also inhibited IFN-γRα, pJak2, and pSTAT1 and upregulated the expression of SOCS1 (Sung and Kim, 2013). Furthermore, the study determined that IVE and its constituents (p-anisaldehyde and *trans*-anethole) inhibited the adhesion of Jurkat T cells to HaCaT cells via ICAM-1 (Sung and Kim, 2013). These anti-inflammatory findings of IVE can directly impact the treatment of IFN-γ-dependent elements of CLE through the demonstrated influence on keratinocyte-T cell interactions (Sung and Kim, 2013).

##### Isodon serra (Maxim.) Kudô [Lamiaceae]

We included two studies that examined the effects that the compounds enmein and oridonin derived from *Isodon serra (Maxim.) Kudô [Lamiaceae]* conferred on lupus-like Balb/C mice. Zhang et al. investigated the effect of four ent-kaurene diterpenoids (enmein, nodosin, lasiodonin, and epinodosin) on the proliferation of murine lymphocytes (Zhang et al., 2005). In this study, the diterpenoids suppressed murine splenic lymphocyte overproduction resulting from Concanavalin A exposure, and more specifically, enmein showed enhanced potency by also interfering with DNA replication in the G1-S stage (Zhang et al., 2005). This study was extended to include the xylene-induced mice tumescence model, which provided further evidence of enmein’s potency by showing cutaneous findings exhibited by depressing swelling in the ear of the murine model and also reducing the expression of IL-2 (Zhang et al., 2005).

Zhou et al. reported that oridonin regulates B-cell activating factor (BAFF) and ameliorates the manifestations of SLE in MRL/lpr mice (Zhou et al., 2013). *In vitro*, oridonin significantly inhibited BAFF expression by suppressing the transcriptional activation of BAFF’s promoter (Zhou et al., 2013). This study concluded that oridonin alleviates SLE symptoms by down-regulating BAFF and reducing the rate of B-cell maturation and differentiation (Zhou et al., 2013). The down-regulation of BAFF is an important finding because BAFF is expressed significantly high in lesional keratinocytes of CLE patients (Wenzel et al., 2018). Although the mechanism by which enmein reduced ear swelling is not discussed, *isodon serra* demonstrates beneficial medicinal value in CLE therapeutics.

##### Nelumbo nucifera Gaertn. [Nelumbonaceae]

This study examined the effect of (S)-Armepavine, an extract of *Nelumbo nucifera Gaertn. [Nelumbonaceae]* (lotus), on T cell proliferation in MRL/MpJ-lpr/lpr mice (Liu et al., 2006). The extract suppressed T-cell proliferation and conferred an ameliorative effect on SLE manifestations by inhibiting splenocyte proliferation and suppressing the expression of IL-2, IL-4, IL-10, and IFN-γ (Liu et al., 2006). Furthermore, (S)-Armepavine reduced glomerular hypercellularity, immune complex deposition, proteinuria, and anti-ds DNA autoantibody production in the murine model (Liu et al., 2006). Considering that Th type 2 cells and local IFN-γ interaction may affect the severity of CLE (Stein et al., 1997), and that (S)-armepavine impaired IL-2/IFN-γ transcripts in human PBMCs (Liu et al., 2006), then a CLE therapeutic can potentially be designed from this compound and exploit a similar mechanism of action.

##### Olea europaea L. [Oleaceae]

Olive oil, a product of *Olea europaea L. [Oleaceae]*, is a widely used ingredient in food preparation. ROS-related diseases and inflammatory processes can be treated with olive leaf extract since it has anti-inflammatory, immunomodulatory, and antioxidant properties (Castejón et al., 2020). A number of phytochemicals derived from olive trees, such as secoiridoids, contain important medicinal properties (Castejón et al., 2020). Moreover, acyl derivatives of natural phenols have shown considerable potential due to their excellent hydrophilic/lipophilic balance, making them potentially beneficial pharmaceutical CLE drug delivery design components (Castejón et al., 2020). These factors combined warrant further research to take advantage of olive oil as a medicine.

##### Paeonia lactiflora Pall. [Paeoniaceae]

Previous studies have demonstrated that Paeoniflorin (PF) from *Paeonia lactiflora Pall. [Paeoniaceae]* (peony) confer anti-allergic and anti-inflammatory effects. This study found that PF suppressed the activation of human T lymphocytes by inhibiting IFN-γ production and the signaling pathway for NF-κB/IκBα and p38 MAPK in mice models with allergic contact dermatitis (ACD) (Wu et al., 2021). The immunosuppressive and anti-inflammatory findings suggest that PF can potentially be used to treat T cell-mediated inflammatory conditions, and these findings can also be translated to the treatment of CLE. A different approach was taken by Wang et al. who studied the impact of PF on the regulation of cytokine production in a murine model of ACD. The study found that PF significantly inhibited cutaneous inflammation and decreased the proliferation of thymocyte (a T cell precursor) in mice with ACD (Wang et al., 2013). Furthermore, PF increased the expression of IL-4 and IL-10 in the serum, as well as thymocyte and splenocyte culture supernatants (Wang et al., 2013). The researchers observed an increase in the severity of dermatitis with an increase in IL-2 and IL-17 expression levels and a decrease in the severity of dermatitis with a decrease in the expression of IL-4 and IL-10 expression levels (Wang et al., 2013). These findings suggest that PF may exert anti-inflammatory effects by regulating cytokine imbalances (Wang et al., 2013) and mitigate the CLE disease process as it is characterized by IFN-regulation of proinflammatory cytokines that orchestrate the B- and T-cell mediated lesional inflammation (Klaeschen and Wenzel, 2016).

The immunosuppressive properties of PF have also been studied in regard to interleukin-1 receptor-associated kinase 1 (IRAK1), which has been associated with the development of SLE in humans (Ji et al., 2018). Specifically, the study investigated the effect of PF on LPS-triggered macrophage activation and the effect of LPS-induced IRAK1-nuclear factor κB (NF-κB) signaling pathways (Ji et al., 2018). Ji et al. found that PF decreased the phosphorylation of IRAK1 and its downstream proteins induced by LPS and inhibited the expression of TNF-α and IL-6 in the MRL/lpr mice (Ji et al., 2018). This study proposed that PF inhibits LPS-induced cell activation by inhibiting the IRAK1-NF-κB pathway in MRL/lpr mouse macrophages, thereby showing potential as a treatment of SLE (Ji et al., 2018) and CLE by extension. This is further supported by research showing IRAK1 as an important component in auto-inflammation processes (Nanda et al., 2016).

##### Paeonia anomala subsp. veitchii (Lynch) D. Y. Hong and K. Y. Pan [Paeoniaceae] and Paeonia lactiflora Pall. [Paeoniaceae]

Radix paeoniae rubra (RPR) is an anti-inflammatory and immune-modulating compound obtained from *Paeonia lactiflora* and *Paeonia veitchii* (Lin et al., 2016). This study found that Prednisone and RPR successfully suppressed the amount of infiltrating inflammatory cells and reduced proteinuria, ergo reducing renal pathology (Wang et al., 2020). Similarly, prednisone and RPR significantly lowered the mRNA expression levels of ICAM-1 and VCAM-1 (Wang et al., 2020). Wang et al. concluded that RPR was as effective as prednisone at reducing ICAM-1, VCAM-1, and PECAM-1 expression in MRL/LPR lupus mice (Wang et al., 2020). These findings reveal the therapeutic potential of RPR in treating SLE and CLE because the expression of adhesion molecules on vascular endothelial cells controls the flow of leukocytes into tissues during an inflammatory response (Malik and Lo, 1996), thus, in order for leukocytes to interact with keratinocytes, ICAM-1 must be involved. ICAM-1 is upregulated by inflammatory cytokines resulting in inflammatory dermatoses characteristic of CLE (Bito et al., 2000) (Wang et al., 2020), and RPR has proven effective in suppressing this process.

##### Pinus pinaster Aiton [Pinaceae]

Pycnogenol, an extract of *Pinus pinaster Aiton [Pinaceae]* (maritime pine), was examined as a treatment option for patients with SLE in a pilot study. In this study, pycnogenol significantly reduced ROS production, apoptosis, p56 (lck) specific activity, erythrocyte sedimentation rate, and also SLEDAI scores (Stefanescu et al., 2001). In a different study, HaCaT cells were used to study the molecular mechanisms underlying Pycnogenol’s effects on T cells and keratinocytes (Bito et al., 2000). Bito et al. found that pycnogenol significantly inhibited IFNγ-induced T cell adhesion (Bito et al., 2000). Additionally, Pycnogenol was shown to significantly inhibit the IFNγ-induced expression of ICAM-1 in HaCaT cells and the IFNγ-mediated activation of STAT1 (Bito et al., 2000). These results suggest that this extract regulates the transcription of inducible ICAM-1 expression (Bito et al., 2000). Pycnogenol may be useful in the development of CLE therapy as an anti-inflammatory.

##### Mentha × Piperita L. [Lamiaceae] + Resveratrol

Pannu and Bhatnagar investigated the effects of Resveratrol and piperine (Mentha × piperita L. [Lamiaceae]) as a treatment and also in prophylactic applications in lupus-like Balb/c mouse models. Resveratrol is a phytoalexin with several pharmacological properties (Pannu and Bhatnagar, 2020a). The polyphenolic compound resveratrol has pharmacological effects and can be found in peanuts, grapes, red wine, and some fruits (Baell and Walters, 2014). The researchers combined resveratrol with piperine (as a bio-enhancer) to treat Balb/c mice, and they found that this led to reductions in IFN-α, IL-6, and TNF-α expression (Pannu and Bhatnagar, 2020a). Furthermore, this treatment reduced proteinuria, creatinine in the urine, and oxidative stress (Pannu and Bhatnagar, 2020a). These findings led to the conclusion that this treatment combination can reduce some measures of lupus morbidity.

Pannu and Bhatnagar further examined whether prophylactic treatment with resveratrol and piperine can prevent lupus-like manifestations in the BALB/c mice (Pannu and Bhatnagar, 2020b).

They found that prophylactic resveratrol and prophylactic piperine were equally efficient in mitigating oxidative stress (enzyme activity of catalase, superoxide dismutase, glutathione peroxidase, and level of reduced glutathione, lipid peroxidation, and reactive oxygen species) (Pannu and Bhatnagar, 2020b). These compounds reduced levels of IL-6 and TNF-α, and piperine administered alone significantly reduced urine creatinine level and proteinuria (Pannu and Bhatnagar, 2020b). Additionally, piperine prophylactic treatment enhanced liver and lung histopathology and decreased pulmonary immune complexes (Pannu and Bhatnagar, 2020b). The study reports that resveratrol decreased the susceptibility of developing a lupus-like disease in the mice, and its effect was not enhanced by piperine (Pannu and Bhatnagar, 2020b). These findings have potential applications in the treatment of CLE as the effect of Resveratrol mimics the inhibition of proinflammatory cytokines such as TNF-α and IFN-α seen in the antimalarials that are currently used in treating SLE and CLE (Alves et al., 2017).

##### Sophora flavescens Aiton [Fabaceae]

This study examined the effect of Sophorae Radix (SR) likely from *Sophora flavescens Aiton [Fabaceae]* on SLE in the NZB/w mice (Ko et al., 2007). SR treatment significantly reduced proteinuria and anti-dsDNA antibodies in serum and glomerular capillaries (Ko et al., 2007). Additionally, SR reduced the expression of IFN-γ in splenocyte culture without affecting IL-4 secretion, leading the researchers to propose that SR can correct deviated Th1/Th2 imbalances in murine models (Ko et al., 2007). This finding suggests SR could be beneficial in treating CLE, considering that CLE is associated with a systemic type I IFN-induced imbalance of Th1/Th2 by shifting towards a Th1-associated chemokine receptor profile (Freutel et al., 2011).

##### Tripterygium wilfordii Hook. f. [Celastraceae]

*Tripterygium wilfordii Hook. f. [Celastraceae]* (TW) has many pharmacological applications due to reported anti-inflammatory, immune modulation, antiproliferative and proapoptotic activity (Liu, 2011). These features make TW effective in treating autoimmune diseases like lupus (Liu, 2011). In this review, we examine three studies using celastrol and tryptolide derived from TW to treat lupus. In one study, Yu et al. investigated whether celastrol can inhibit the formation of neutrophil extracellular traps (NET) induced by inflammatory stimuli associated with SLE (Yu et al., 2015). This study found that celastrol can completely inhibit neutrophil oxidative burst and NET formation induced by TNFα, ovalbumin:anti-ovalbumin immune complexes (Ova IC), and immunoglobulin G (IgG) (Yu et al., 2015). This study also found that celastrol can downregulate the SYK-MEK-ERK-NFkB signaling cascade, thereby inhibiting neutrophil oxidative burst and NET formation induced by different inflammatory stimuli (Yu et al., 2015). In a different study using Trex1−/− mice, celastrol inhibited interferon regulatory factor 3 (IRF3) activation leading to a reduction of the interferon response triggered by cytosolic nucleic acids, autoantibody production, and excessive T-cell activation (Liu et al., 2020).

Our search revealed two studies examining the effects of triptolide. One study focused on the effects on MRL/lpr mice (Huang X. et al., 2018), and another study examined the effect of triptolide on the expression of P40 gene in APCs (Zhang and Ma, 2010). Triptolide treatment improved skin damage, decreased serum levels of IFN-γ and IL-10, improved renal histopathologic characteristics of the mice, and downregulated the mRNA level of TLR9, TLR4, and NF-κB (Huang X. et al., 2018). The investigation by Zhang and Ma into the molecular mechanism of triptolide inhibitory effect on the expression of the p40 gene in APCs revealed that Triptolide could activate the transcription of C/EBPalpha, and phosphorylation of Ser21 and Thr222/226, which are critical for C/EBPalpha inhibition of p40 (Zhang and Ma, 2010). Additionally, this study found that the activation of C/EBPalpha by triptolide is affected by upstream kinases ERK1/2 and Akt-GSK3beta (Zhang and Ma, 2010). This is an important finding considering celastrol can downregulate the signaling cascade of the SYK-MEK-ERK-NFkB (Yu et al., 2015), and the downregulation of IL-12 p40 (which correlates negatively with lupus SLE activity), which ultimately contributes to excessive IL-10 (positively correlated with lupus pathology) (Liu et al., 1999). The implications of these findings are profound in that TW’s extracts triptolide and celastrol collectively confer broad immunomodulatory effects. These extracts in treatment involve neutrophils, NETs, TLRs, and IFN-dependent pathways that have a direct effect on SLE and CLE disease process.

##### Aconitum lamarckii Rchb. ex Spreng. [Ranunculaceae]

*Aconitum lamarckii Rchb. ex Spreng. [Ranunculaceae]* (Wolf’s-bane’s) main ingredient, aconitine, has immunomodulatory properties that have applications in treating autoimmune diseases (Li et al., 2017). Li et al. explored the effect and mechanisms of aconitine on the treatment of pristane-induced murine model of SLE. This study found that aconitine decreased elevated blood leukocyte counts, prostaglandin E2 (PGE2), IL-17a, IL-6, serum anti-dsDNA antibody, and IgG deposit in glomerular (Li et al., 2017). Li et al. concluded that aconitine could inhibit autoimmune disease progression and ameliorate lesions characteristic of SLE pathology (Li et al., 2017). It is known that PGE2 is an important mediator of inflammation based on its inhibition of the activation of T cells (Sakata et al., 2010). PGE2 can also trigger Th1 and Th17 differentiation in certain circumstances through the elevation of cAMP via PGE receptors EP2 and EP4 (Sakata et al., 2010). This is important because Th1 and Th17 cells are among the adaptive immune cells involved in the pathology of CLE (Achtman and Werth, 2015). Aconitine’s ability to disrupt the action of PGE2 suggests its potential as a CLE therapeutic.

#### 3.3.4 Fungi-*Ganoderma lucidum (LZ)*

We identified one study of Ganoderma lucidum (LZ), a relative of Reishi mushroom, with San-Miao-San (SMS) extract, a “four marvels” herbal powder comprised of 33.3% Yi Yi Ren, 16.6% Cang Zhu, 16.6% Huai Niu Xi, and 33.3% Huang Bai. Cai et al. tested LZ extract with SMS in MRL/lpr mice. LZ-SMS significantly reduced concentrations of anti-dsDNA antibodies, induced Treg and Breg formation, reduced IL-21, IL-10, and IL-17A in serum, and increased IL-2 and IL-12p70 in serum (Cai et al., 2016). It is unclear how much of this effect was due to LZ versus SMS.

#### 3.3.5 Bacteria

##### Lactobacilli

Similar to regulating the gut microbiota, *lactobacilli* have the potential to modulate immune responses. An *in vivo* study evaluated the effects of *lactobacillus* species on Th17 cells and their related mediators in a pristane-induced BALB/c mice model of SLE (Mardani et al., 2019). Subjects that were assigned the probiotics and prednisolone treatment groups had delayed onset of SLE with a significant reduction in antinuclear antibody (ANA), anti-double-stranded DNA (anti-dsDNA), anti-ribonucleoprotein (anti-RNP), and severity of lipogranuloma lesions compared to positive controls (Mardani et al., 2019). The results also revealed a significant reduction in IFN-γ and IL-17 levels among the same treatment groups (Mardani et al., 2019). Furthermore, there was a significant decrease in the CD4+IL*17+, CD4+IFN+, and CD8+IFN*+ populations in groups that received probiotics or prednisolone (Mardani et al., 2019). The study was rated a medium risk of bias and was conducted on mice, which limits its applicability to humans. Despite this, the presenting data suggest that probiotics may be useful as adjunctive therapy for reducing SLE severity.

#### 3.3.6 Herbal Formulas

Two studies were performed using herbal formulas to treat SLE patients (formulation information for herbal formulas is provided in Supplementary Table S3). Zhong et al. performed a single-blind randomized control study of Zi Shen Qing (ZSQ), which is a mixture of Radixastragali, Rehmannia glutinosa libosch, Fructus Corni, Paeonia lactiflora, Herba Hedyotis Diffusae, and Cortex Moutan Radicis. ZSQ preparation was used to treat SLE patients with SLEDAI of 5–14. The authors reported a significant reduction in SLEDAI-2000, improved tapering of corticosteroids, and reduced incidence of flare in treated patients (Zhong et al., 2013). Liao et al. performed a double-blind, randomized controlled trial of Dan-Chi-Liu-Wei combination (DCLWC) as an add-on therapy with prednisolone to treat SLE patients with SLEDAI of 2–12. Adding-on DCLWC to conventional therapy was safe and had a borderline effect in decreasing disease activity, but it was not possible to taper the steroid dosage after 6 months (Liao et al., 2011). These studies had a low risk of bias owing to being randomized controlled trials.

An additional five studies performed experiments testing herbal formulas on MRL/lpr mice. Virtually all of these studies were conducted in one cohort of animals, leading to a moderate to high risk of bias. Two of these studies specifically impacted skin disease. Kanauchi et al. tested Sairei-to, also called Kampo, which is an herbal formula consisting of the following 12 ingredients: bupleuri radix, pinelliae tuber, scutellariae radix, ginseng radix, glycyrrhizae radix, zizyphi fructur, zingiberis rhizoma, atractylodis lanceae rhizoma, polypourus, alismatis rhizoma, hoelen and cinnamoni cortex. Mice treated with this formula exhibited reduced IgG deposition at the dermo-epidermal junction (DEJ) in the skin, as well as reduced titers of anti-DNA antibodies, rheumatoid factor, and reduced lymphoproliferation (Kanauchi et al., 1994). Zhu et al. tested Naja Naja Atra Venom (NNAV), which contains cobra venom factor, cardiotoxins, and neurotoxins. NNAV protected against skin erythema and proteinuria, and reduced glutamate pyruvate transaminase, creatinine kinase, circulating globulin, anti-dsDNA antibody, and inflammatory cytokines IL-6 and TNF-α (Zhu et al., 2014). Lymphadenopathy and renal injury were also reduced in MRL/lpr mice treated with NNAV. Serum C3 levels were increased, indicating disease improvement via less consumed C3 (Ricker et al., 1991). C3 level measurement in SLE patients is a complicated biomarker (Birmingham et al., 2010), potentially warranting further study into potential detrimental effects of increased C3 production, as opposed to reduced C3 consumption, following NNAV.

Three studies demonstrated improvement of inflammation in MRL/lpr mice following treatment with herbal formulas. Lee et al. used Longdan Xiegan Tang (LXT) formula, which is comprised of 10 ingredients: Gentiana rigescens Franch, Scutellaria baicalensis Georgi, Gardenia jasminoides Ellis, Alisma orientalis (Sam.) Juzep, Clematis Montana Buch.-Ham., Plantago asiatica L, Angelica sinensis (Oliv.) Diels, Rehmannia glutinosa Libosch, Bupleurum chinense DC, and Glycyrrhizae uralensis Fisch. Treatment with LXT reduced IFNg, TNF, anti-ds DNA antibody, and increased splenic regulatory T cell populations (Lee and Chang, 2010). In addition, LXT improved kidney glutathione levels and decreased oxidative stress in MRL/lpr mice. The remaining two studies tested Jieduquyuziyin prescription (JP). JP is comprised of the following ten herbs: Rehmanniae glutinosa (radix), Trionycis carapax, Scleromitrion diffusum, Hedyotis diffusa, Paeonia anomala subsp. Veitchii, Centella asiatica, Paeonia × suffruticosa Andrews, Citrus medica L., Actaea cimicifuga L. (syn. Cimicifuga foetida L.), Glycyrrhiza uralensis Fisch. Ex DC. Ji et al. tested both Sprague Dawley rats in addition to MRL/lpr mice. Ji et al. found that JP treatment inhibited phosphorylation of IRAK1 and its downstream proteins induced by LPS (Ji et al., 2019b, 2020). JP also inhibited the expression of TNF and IL-6 and reduced peritoneal macrophage activation. A second study by Shui et al. found that JP treatment in MRL/lpr mice reduced Th17 cell responses, including inhibition of IL-17 and RORgT expression, and inhibited CaMK4 expression (Shui et al., 2015).

Studies using herbal formulas provide challenges to data interpretation for several reasons. First, compounds can have interactions that are additive, synergistic, negative, or null. Second, it may be that a compound’s metabolite that is generated in the body following processing or degradation provides the therapeutic benefit. JP can lead to the generation of 13+ metabolites (Ding et al., 2014). Future studies for these herbal formulas are warranted, particularly for identifying potential synergistic combinations of compounds that minimize unwanted side effects.

#### 3.3.7 Other Natural Formula-Toxins

Zhu et al. tested Naja Naja Atra Venom (NNAV), which contains cobra venom factor, cardiotoxins, and neurotoxins in MRL/lpr mice. NNAV protected against skin erythema and proteinuria, and reduced glutamate pyruvate transaminase, creatinine kinase, circulating globulin, anti-dsDNA antibody, and inflammatory cytokines IL-6 and TNF-α (Zhu et al., 2014). Lymphadenopathy and renal injury were also reduced in MRL/lpr mice treated with NNAV. Serum C3 levels were increased, indicating disease improvement via less consumed C3 (Ricker et al., 1991). C3 level measurement in SLE patients is a complicated biomarker (Birmingham et al., 2010), potentially warranting further study into potential detrimental effects of increased C3 production, as opposed to reduced C3 consumption, following NNAV.

#### 3.3.8. Compounds

##### Macro, Nutraceuticals, and Micronutrients

A systematic review evaluated the clinical and preclinical scientific evidence of diet and dietary supplementation that either ameliorate or exacerbate SLE symptoms (Islam et al., 2020). Islam et al. reported that a diet high in fiber, polyunsaturated fatty acids, vitamins, minerals, and polyphenols contain sufficient macronutrients, and micronutrients can modulate inflammation and the immune system of SLE patients (Islam et al., 2020). While the scope and conclusions of this study are rather broad, they are applicable to CLE since it is a manifestation of SLE and can develop without systemic involvement. In fact, in a study by Berthier et al., there were no major transcriptional differences between CLE only and SLE-associated CLE lesions (Berthier et al., 2019).

##### Taxifolin

Bito et al. investigated the effect of plant flavonoids on intercellular adhesion molecule-1 (ICAM-1) expression in human keratinocytes. Taxifolin was selected for its potency and its potential to inhibit IFNγ-induced ICAM-1 protein and mRNA expression in human keratinocytes (Bito et al., 2002). The study found that taxifolin inhibited the activation of STAT1 and the phosphorylation of JAK1, thereby affecting the expression of ICAM-1 at the transcriptional level (Bito et al., 2002). Furthermore, taxifolin inhibited the expression of ICAM-1 induced by IFN-γ in an experimental human skin, suggesting that taxifolin may have therapeutic potential in pathological skin conditions caused by enhanced adhesion and inflammation (Bito et al., 2002). The study concluded that the JAK-STAT pathway might be the site of molecular action for taxifolin (Bito et al., 2002), and by extension, a potential pathway that a CLE therapeutic can be designed to exploit.

##### N-Acetylcysteine

Studies show that microbiome composition and function have effects on the pathogenesis of autoimmune diseases (ADs). Wang et al. examined the role of the gut microbiome and host responses in SLE pathogenesis using female C57BL/6, MRL^+/+^, and MRL/lpr mouse models with varying levels of disease progression (Wang H. et al., 2021). The researchers found that N-acetylcysteine treatment decreased the Rikenellaceae population; increased the population of Akkeransiaceae, Erysipelotrichaceae, and Muribaculaceae; and improved Turibaculaceae attenuation. This increase in gut microbiota dysbiosis was linked to an increase in the oxidative stress of the gut, barrier dysfunction, inflammatory responses, and systemic autoimmunity (Wang H. et al., 2021). The skin microbiota is not well-studied, though one study has reported increased *Staphylococcus aureus* in CLE lesions (Sirobhushanam et al., 2019). We can posit that if systemic autoimmunity is correlated with a disruption in gut microbiota and correction of dysbiosis can have a positive impact on disease course, then cutaneous manifestations of autoimmunity can be mitigated or reversed with skin pre- or probiotics to restore the microbiome.

##### Sodium Butyrate

Gut microbiota dysbiosis strongly influences the onset and development of systemic lupus erythematosus (SLE). Several studies have demonstrated microbiota-derived butyrate’s effectiveness in ameliorating SLE (He et al., 2020). This study used MRL/lpr lupus-prone mice to examine the roles of butyrate on gut microbiota in SLE (He et al., 2020). The researchers found that Butyrate supplementation ameliorated gut microbiota dysbiosis and renal histopathologic changes in lupus-prone MRL/lpr mice, therefore showing potential as SLE treatment (He et al., 2020). Based on this data, it is clear that the gut microbiome and host interactions influence SLE disease manifestation, which can be applied to CLE, given their similarities. This study had an overall low risk of bias; however, more research in this area is needed to identify the role of gut microbiome dysbiosis in CLE.

##### Arsenic Trioxide and Tetra-Arsenic Tetra-Sulfide

Arsenic trioxide (ATO), used for treating acute promyelocytic leukemia, has shown the potential to treat lupus-like disease processes in animal models (Hamidou et al., 2021). In a phase IIa clinical study, researchers evaluated the efficacy and safety of intravenous ATO treatment for patients with active SLE (Hamidou et al., 2021). The outcome of this study shows that ATO as a complementary treatment resulted in a decrease in corticosteroid dosage from 11.25 mg/day at baseline to 6 mg/day at week 24 (Hamidou et al., 2021). The researchers concluded that ATO offers a good safety profile and is efficacious in treating patients with SLE (Hamidou et al., 2021).

Zhao et al. cited the findings of the ATO clinical study by Hamidou et al. in their decision to explore the compound Tetra-arsenic tetra-sulfide (As4S4). Tetra-arsenic tetra-sulfide (As4S4) is used in TCM and mainstream medical oncology in treating acute promyelocytic leukemia with milder side effects than ATO (Zhao et al., 2013; Zhu et al., 2013). This study examined As4S4 to determine the side effects and inflammatory parameters affected by As4S4 on the lupus-prone BXSB mice model (Zhao et al., 2013). As4S4 treatment conferred improvement on monocytosis in the spleen and decreased serum levels of IL-6 (Zhao et al., 2013). Several improvements in systemic findings were also identified, i.e., the model mice’s skin, liver, and renal disease (Zhao et al., 2013). As4S4 treatment also suppressed immune complex deposition, mesangial proliferation, and infiltration of inflammatory cells in kidneys and livers (Zhao et al., 2013). This study concluded that As4S4 selectively suppresses cutaneous lupus in BXSB mice (Zhao et al., 2013). The cutaneous-related findings in this study suggests direct application of As4S4 to CLE treatment, though further experimentation is necessary to clarify these findings.

##### Ethyl Pyruvate

Several mesenchymal stem cell (MSC) defects have been observed in SLE patients, including impaired growth, senescence phenotype, and immunomodulatory functions (Ji et al., 2019a). Treatment with ethyl pyruvate (EP) improved the clinical signs associated with lupus nephritis in MRL/lpr mice and elongated the survival of the mice (Ji et al., 2019a). This study found that targeting HMGB1 reverses the senescent phenotype exhibited by bone marrow stromal cells in the model mice (Ji et al., 2019a). In support of this study, MSCs have been shown to synthesize trophic mediators, such as growth factors, cytokines, macrophage colony-stimulating factor, IL-6, IL-11, IL-15, stem cell factor, and VEGF-involved in hematopoiesis regulation, cell signaling, and immunity modulation (Cras et al., 2015). Together, these features indicate that MSCs may be used to treat autoimmune and autoinflammatory symptoms associated with SLE and CLE.

## 4 Future Directions

Plant-derived antimalarials and immunosuppressants are currently used in CLE treatment regimens. This study sought to identify potential compounds derived from plants and natural products that could be repurposed for CLE treatment. Although the studies included in this review are not conclusive and require further research, we can propose that the findings have ethnopharmacological relevance on the development of CLE prophylactics, treatment, and disease managing medications. Different formulations, including topical treatments, of existing therapies that have proven clinical significance in the treatment of SLE, could be developed for skin-limited lesions or as adjuncts for the existing CLE therapies. Topical treatments generally have better safety profiles and could ameliorate the toxicity concerns of some of the reviewed compounds by limiting systemic absorption.

Nutrition has long been recognized in improving the course of lupus erythematosus. Macro and micronutrients, including flavonoids and polyphenols derived from plants and diets rich in polyunsaturated fatty acids, significantly impact lupus patients’ quality of life, suggesting a beneficial or synergistic effect of diet in symptom management. We identified vitamin D as a potentially important bioactive molecule for CLE patients through our search. It is important to note that synthetic vitamin A derivatives (retinoids) and vitamin B/folic acid analogs (metformin) are also used to treat rheumatic diseases, including CLE (Kuhn et al., 2011). Patients self-report improved symptoms or symptom management with improved diet (Charoenwoodhipong et al., 2020). Future studies examining potential additive or synergistic effects of vitamins/diet on CLE disease damage and activity in combination with anti-inflammatories are warranted.

Certain compounds and extracts demonstrated efficacy specifically for skin disease. These included *Tripterygium wilfordii Hook. f.*, fish oil, and aconitine, Tween-20 Perna, vitamin D. Other compounds we identified were anti-inflammatory and exerted effects on pathways previously targeted in lupus, such as IFN-dependent and signaling pathways for cytokines, JAK-STAT, B cell intrinsic signaling, NFkB/lkBa, and P38 MAPK. These compounds included extracts from *Tripterygium wilfordii Hook. f.*, *Camellia sinensis (L.) Kuntze, Artemisia annua L., Curcuma longa L.,* and *Paeonia lactiflora Pall*. These findings are promising because closely related plants may confer similar effects, therefore impacting the availability of viable raw materials. Further research will be beneficial in verifying this proposition.

Ji et al.’s study of JP and PF- mediated inhibition of IRAK1 is of particular interest to our group, as we recently identified IRAK1 as a potential treatment target in CD45^+^ immune cells in discoid lupus erythematosus (DLE) lesional skin using spatial transcriptomics (Haddadi et al. submitted, GSE182825). Therefore, we posit that active ingredients from JP and PF that target IRAK1 may be promising for further pharmaceutical development.

Certain compounds we identified are notably toxic at high doses, including arsenic derivatives. *Tripterygium wilfordii Hook. f.* can also be detrimental to patients when its safety is not properly controlled during preparation. This is because it has a narrow therapeutic index and may result in unavoidable side effects (Ru et al., 2019). Methotrexate is also genotoxic, serving as a chemotherapeutic that limits lymphocyte proliferation. Dosing for toxins, including NNAV would need to be performed carefully to avoid unwanted side effects. Allergies are another potential, though this is a risk with virtually any drug, food, or biologic medication.

Patient education is another important component of utilizing herbal supplementation, TCM, and other approaches, as these active compounds may interact with a patient’s current medication list. The efficacy and safety of medicinal extracts are influenced by their purity and concentration of active ingredients relative to the excipients during preparations; when much attention is not paid to these factors, a lack of clinical response or side effects ranging from mild symptoms to life-threatening ones can occur.

We would like to highlight that ancient cultures and indigenous peoples have used natural compounds to treat various ailments, passing down traditional wisdom through generations (Salmon, 1996). This wisdom may be missing from the peer-reviewed scientific literature. Cultural appropriation has monetized these approaches to produce pharmaceutical-grade medications ultimately. It is understood that the development of pharmaceuticals requires a certain level of funding for purification/synthesis, preclinical and clinical testing to prove mechanism, safety, dose, and superiority to previous standards of care. Furthermore, given that patients can have severe disease that is not fully controlled through herbalism, patients may ultimately need a stronger or more purified compound to achieve disease control or remission. In our modern society, patients are exposed to a myriad of environmental triggers through climate change and pollution, contributing to their disease severity. Thus, we would encourage future research into the plant extracts and natural compounds we identified as potential novel CLE treatments, but to caution scientists to do so in a way that will not decimate the land of the people who cultivate medicinal plants, not make the end product unaffordable to patients, and not disregard the work of traditional healers.

### 4.1 Limitations

The main challenge of this project was producing an exhaustive list of plant extracts and natural products in our search strategy. For instance, we included all general plant terms and then those we suspected would yield usable data, but we could not feasibly account for all plant names, extracts, and products in the search. Our search strategy yielded many studies regarding synthetic immunomodulators, such as JAK inhibitors. The studies focused on components that were not directly in line with our inclusion criteria, i.e., the sourcing criteria and the respective use in the treatment of lupus. Nonetheless, we were successful in discovering medicinal plant extracts and natural compounds we had not yet considered as potential novel treatments.

Additionally, several studies we extracted lacked mechanistic experimentation, which could contribute to bias in the interpretation of efficacy and reported results. Many mouse studies that used MRL/lpr mice were only conducted once; this is likely because the mice need to be aged to test lupus disease states. However, experimental n of 1 confers a high risk of bias, as it is unclear whether the results hold true over time. Studies using herbal formulas provide challenges to data interpretation for several reasons. First, compounds can have interactions that are additive, synergistic, negative, or null. Second, it may be that a compound’s metabolite that is generated in the body following processing or degradation provides the therapeutic benefit. JP can lead to the generation of 13+ metabolites (Ding et al., 2014), underscoring the need for further research to discern how metabolites impact lupus disease. Future studies for these herbal formulas are warranted, particularly for identifying potential synergistic combinations of compounds that minimize unwanted side effects.

Another important consideration is that different compounds may be more efficacious for specific clinical subtypes of CLE, and for CLE that exists with or without systemic involvement. This would need to be assessed further through preclinical studies and clinical trials, and is therefore beyond the scope of this systematic review at this time.

### 4.2 Potential Impacts on Photosensitivity

It is important to note that *Hypericum perforatum L. [Hypericaceae]* (St. John’s wort), which we identified in our study, and *Ginko biloba* L., which was not captured in our search but is a commonly used herbal supplement, are photosensitizers. These may have a negative impact on CLE disease if patients use these supplements and are exposed to UV light without proper photoprotection (Levine 1990 from Wisconsin Department of Public Health). Other compounds identified in our search that could cause photosensitivity include quercetin, which was shown to be phototoxic *in vitro* in HaCaT cells (Rajnochová Svobodová et al., 2017), and SMS preparation, which includes components from *Phellodendron amurense Rupr.* [Rutaceae]. Rutaceae used in perfumes, flavoring, and spices can cause photosensitivity (Levine 1990 from Wisconsin Department of Public Health).

However, antimalarials, which are mainline therapies used to treat CLE, and synthetic vitamin A derivatives (retinoids), which are used to treat rheumatic diseases, can also cause photosensitivity (Moore and Hemmens, 1982; van Weelden et al., 1982; Blakely et al., 2019). Further, pharmaceutical agents synthetic and naturally derived alike may interact with each other to cause photosensitivity. Therefore, counseling patients on sun avoidance and protection as well as disclosure of all medications and supplements used is paramount in management of CLE. Another option may be to find safe combinations of treatments to control photosensitivity, for example by combining a photoprotective supplement like *Phlebodium aureum (L.) J. Sm*. [Polypodiaceae] extract (Alonso-Lebrero et al., 2003; Prado et al., 2018; Segars et al., 2021) with an anti-inflammatory agent as was performed in a case report of subacute CLE (Breithaupt and Jacob, 2012).

Of the remaining compounds we identified, some exhibit evidence of photoprotection including fish oils and fatty acids (Rhodes et al., 1995; Rhodes and White, 1998; Pilkington et al., 2011; Huang TH. et al., 2018). *Tripterygium wilfordii Hook. f*. exhibited photosensitizing activity against bacteria and fungi, but has no reported evidence of photosensitizing activity in mammalian cells (Alam et al., 2021). This may be a beneficial aspect that should be studied in the context of CLE disease: dysbiosis occurs in CLE (Sirobhushanam et al., 2019) and targeting microbial cells but sparing the host cells could be beneficial. *Curcuma longa* L. (curcumin, turmeric) is also being examined for anti-microbial photosensitization (Yang et al., 2020), however there is evidence of potential negative impacts on mammalian cells, as people can experience pigmentation differences following UV exposure (Napolitano, 2019). Last, several compounds we identified are sensitive to oxidation and degradation caused by exposure to UV light, including olive oil (derived from *Olea europaea* L. [Oleaceae]), *Epimedium alpinum L. [Berberidaceae]* extracts, and resveratrol, potentially making extraction and storage of active compounds difficult (Khan and Shahidi, 1999). Notably, olive oil itself does not impact phototherapy and is unlikely to cause photosensitivity by itself (Fetil et al., 2006; Zeb et al., 2008; Akarsu et al., 2018).

## 5 Conclusion

It is clear that more clinical and immunologically heterogeneous therapies are needed for lupus and CLE. Historically, plant-derived compounds have been a source of inspiration for the development of cutaneous disease treatment, like immunosuppressants and antimalarials. In this systematic review, we analyzed a number of experimental and epidemiological studies concerning natural compounds/plant extracts in order to assess their effectiveness and immunological effects. Despite the lack of conclusive evidence in many of these studies, it remains clear that many of these substances have anti-inflammatory and immunomodulatory properties that may be applied to CLE treatment. There was an improvement in inflammatory markers, cytokine levels, immunological pathways, skin lesions severity, assessment scores, and patient quality of life in several studies identified in our literature search. As scientists continue to look for better ways to treat CLE, the compounds we have identified in this systematic review warrant further studies for the possibility of harnessing new active ingredients for the treatment of CLE.

## Data Availability

The original contributions presented in the study are included in the article/Supplementary Material, further inquiries can be directed to the corresponding authors.
