# Supplementary material for: Medicinal Plant Extracts and Natural Compounds for the Treatment of Cutaneous Lupus Erythematosus: A Systematic Review
Source: Front Pharmacol. 2022 Mar 31;13:802624. doi: 10.3389/fphar.2022.802624 (PMC9008762; doi:10.3389/fphar.2022.802624)
Supplement: Supplementary file 3 [file Table1.docx]

**Supplementary Table 1**

**Full Ovid Medline Search Strategy**

Full Ovid Medline Search Strategy

1. exp Lupus Erythematosus, Cutaneous/ or ((cutaneous adj3 lupus) or (lupus adj3 erythemat*) or (skin adj3 lupus) or (discoid* adj3 lupus) or (local adj3 lupus) or pannicul* or (lupus adj3 profundus)).ti,ab.
2. exp Plants/ or (plant or plants or plantae or tree or trees or vegetable* or coffee* or oil* or extract* or (Bach adj3 rescue adj3 remed*)).ti,ab.
3. exp Plant Structures/ or (bulb* or sprout* or twig* or flower* or anther* or blossom* or carpel* or floral or floret* or gynaecium or gynoecium or nectar* or ovule* or petal* or pistil* or pollen or stamen* or fruit* or berry or berries or (legume adj3 pod*) or nut or nuts or seed* or bark or leaf or leaves or ((conifer* or pine) adj3 needle*) or frond* or shoot or shoots or stem or stems or branch* or wood* or shrub* or root or roots or stalk or stalk* or tuber or tubers).ti,ab.
4. exp Fungi/ or (fungi* or fungus* or mold* or moss* or agaricales or agaricaceae or mushroom* or toadstool* or lichen*).ti,ab.
5. Teas, Medicinal/ or Herbal Medicine/ or Medicine, Chinese Traditional/ or (tea or teas or (barley adj3 water*) or tisane* or herb* or botanic* or (La'au adj3 Lapa'au) or (Laau adj3 Lapaau) or phyto-medicine or phytomedicine or (Chinese adj3 medicin*) or (Chung adj3 I adj3 Hsueh) or (fang adj3 ji adj3 fen adj3 lei) or (fangji adj3 fenlei) or fangjifenlei or (traditional adj3 tongue) or (Zhong adj3 Yi adj3 Xue) or (natural adj3 (product* or compound*))).ti,ab.
6. exp Bacteria/ or (bacteri* or eubacteri* or schizomycetes).ti,ab.
7. or/2-6
8. exp Angelica/ or (angelica* or archangelica* or ashitaba or (dang adj3 gui) or (dong adj3 quai) or (ligusticum adj3 acutilobum) or (peucedanum adj3 decursivum) or (tang adj3 kuei) or (tang adj3 kwei) or (yamato adj3 tohki)).ti,ab.
9. exp Callicarpa/ or (callicarpa or beautyberr*).ti,ab.
10. exp Camellia Sinensis/ or ((camellia or thea) adj3 sinensis).ti,ab.
11. Chamomile/ or (chamomile* or camomile* or chamomilla*).ti,ab.
12. Curcuma/ or (curcuma* or tumeric* or turmeric*).ti,ab.
13. ((epigallocatechin adj3 gallate) or ECGC).ti,ab.
14. Garlic/ or (garlic* or (allium adj3 sativum) or carisano).ti,ab.
15. Ginger/ or (ginger* zingiber* or zinziber*).ti,ab.
16. exp Mentha/ or (mentha* or mint* or horsemint* or peppermint* or pennyroyal* or spearmint*).ti,ab.
17. exp Piper/ or (piper* or pepper* or futokadsura or betel or cubeb or pippali or kava*).ti,ab.
18. Sinomenium/ or (sinomeni* or (qing adj3 feng adj3 teng*)).ti,ab.
19. Triptergium/ or (tripterygium* or (leigong adj3 teng*) or (lei adj3 gong adj3 teng*) or leignongteng or thundergod or celastrol or tripterin*).ti,ab.
20. Cinnamomum Zeylanicum/ or ((cinnamomum adj3 (verum or zeylanicum)) or (cinnamomi adj3 cortex) or cinnamon*).ti,ab.
21. Olive Oil/ or ((olive adj3 oil*) or (oleum adj3 olivarum) or oleumolivarum).ti,ab.
22. or/8-21
23. Janus Kinase Inhibitors/ or ((janus adj3 kinase adj3 inhibitor*) or (jak adj3 inhibitor*)).ti,ab.
24. Antimalarials/ or (antimalarial* or anti-malarial* or antipaludean or antiplasmodic).ti,ab.
25. exp Artemisinins/ or (artemisin* or arteannuin or (ching adj3 hao adj3 su) or chinghaosu or ginghaosu or nsc369397 or qinghaosu or quinghaosu or quinhaosu or artemether or aids-008344 or aids008344 or artemetero or (artemininelactol adj3 methyl adj3 ether) or artenam or arthemether or cgp 56696 or cgp56696 or (dihydroartemisinin* adj3 methyl adj3 ether) or (dihydroquinghaosu adj3 methyl adj3 ether) or (methyl-dihydroartemisinine adj3 sm adj3 "224") or o-methyldihydroartemisinine or paluther or coartem* or "co artem" or cgp56697 or cgp 56697 or coa566 or "coa 566" or riamet or artesunat* or arsumax or (artesunic adj3 acid*) or (succinyl adj3 dihydroartemisinin) or (dihydroartemisinin* adj5 succinate) or malacef or malartin or plasmotrim or sm804 or "sm 804").ti,ab.
26. exp Chloroquine/ or (chloroquin* or a-cq or amokin* or anoclor or aralan or aralen* or arechin* or arequin* or arthrochin* or arthroquine or artrichin* or artiquine or avloclor or avoclor or bemaphat* or bemasulph or bipiquin or cadiquin or chemochin* or chingamin* or chloraquine or chlorochin* or chlorofoz or chlorquin* or choloquin or (choroquine adj3 sul*) or cidanchin or (clo-kit adj3 junior) or clorichin* or cloroiquine or chlorochina or delagil or delagyl or dichinalex or diclokin or diquinalex or diroquine or emquin or genocin or gontochin* or gontoquine or heliopar or imagon or iroquine or khingamin or klorokin* or lagaquin or malaquin or malarex or malarivon or malaviron or maliaquine or maquine or mesylith or mexaquin or mirquin or nivachine or nivaquin* or p roquine or quinachlor or quingamine or repal or resochen* or resochin* or resoquin* or reumachlor or roquine or rp3377 or rp 3377 or sanoquin* or silbesan or siragan or sirajan or sn7618 or sn 7618 or solprin* or tresochin* or tresoquine or trochin* or troquine or w7618 or w 7618 or win244 or win 244 or hydroxychloroquine or chloroquinol or ercoquin or hydrochloroquine or hydrocloroquine or hydroxychlorochin or oxychloroquine or oxychlorochin or quensyl or sn 8137).ti,ab.
27. Quinidine/ or (quinidine or adaquin or chinidin* or conchinine or conchonine or conquinine or pitayine or quinidate or quiniday or quinora or quinidex or quincardine).ti,ab.
28. Quinine/ or (quinin* or 6 methoxycinchonine or biquinate or chinimetten or chinin* or kinine or kinnine or legatrim or myoquine or quinimax or quinamm or quinbisan or quinbisul or quindan or quinoctal or quinson or quinsul or reductor or sn 359 or strema or surquina or quinimax).ti,ab.
29. ((dichloroallyl adj3 lawsone) or dichlorolapachol or nsc 126771 or (acetylsalicylic adj3 acid adj3 hydroxychloroquine adj3 (sulfate or sulphate)) or (aspirin adj3 hydroxychloroquine adj3 (sulfate or sulphate)) or planolar or ((artemisinic or arteannuic or artemisic) adj3 acid*) or artemisone or artemifone or bay 44 9585 or bay 449585 or bay44-9585 or cinchonidine or (alpha adj3 quinidine) or cinchonine or la 40221 or girolline or giracodazole or giradazole or nsc627434 or nsc 627434 or rp49532 or rp49532a or rp 49532 or rp 49532a or lapachol or greenhartin or (lapaphic adj3 acid*) or nsc-11905 or (taiguic adj3 acid*) or tecomin).ti,ab.
30. or/24-29
31. 7 or 22 or 23 or 29
32. 1 and 31
